# Supplementary material for: Distributed electrified heating for efficient hydrogen production
Source: Nat Commun. 2024 May 8;15:3868. doi: 10.1038/s41467-024-47534-8 (PMC11078997; doi:10.1038/s41467-024-47534-8)
Supplement: Supplementary file 1 — Supplementary Information [file 41467_2024_47534_MOESM1_ESM.pdf]

Supplementary Information

Distributed electrified heating for efficient hydrogen production

Hanmin Yang[1], Ilman Nuran Zaini[1], Ruming Pan[2], Yanghao Jin[1], Yazhe Wang[1], Lengwan Li[3], , José Juan Bolívar Caballero[1], Ziyi Shi[1], Yaprak Subasi[4], Anissa Nurdiawati[5], Shule Wang[6,7], Yazhou Shen[8], Tianxiang Wang[9], Yue Wang[9], Linda Sandström[10], Pär G. Jönsson [1], Weihong Yang[1], Tong Han[1,*]

Correspondence to: tongh@kth.se; +46 725660043

This file includes:

Materials and Methods
Sample characterization
Electrochemical performance test
Computational fluid dynamic (CFD) simulations
Electrified methane pyrolysis process and techno-economic assessment
Supplementary discussion 1-4
Figs. S1 to S29
Tables S1 to S11

Table of Contents

1 Materials and Methods 7

1.1 CH_4 pyrolysis using wood carbon monolith reactor 7

1.1.1 Fabrication of the wood carbon monolith 7

1.1.2 CH_4 pyrolysis test 7

1.1.3 CH_4 conversion, H_2 yield, and C_2H_2 yield calculation 9

1.1.4 CH_4 pyrolysis over commercial graphite crucible 10

1.2 Catalytic CH_4 dry reforming using a metal monolith reactor 10

1.2.1 Preparation of the metal monolith reactor with Ni/MgO catalyst washcoat 10

1.2.2 Catalytic CH_4 dry reforming test 11

1.2.3 CH_4 and CO_2 conversion, syngas production capacity, H_2 and CO yield calculation 11

2 Sample characterization 12

3 Electrochemical performance test 13

4 Computational fluid dynamic (CFD) simulations 14

4.1 Geometry and heating scenarios definition 14

4.2 Boundary equations 15

4.3 Fluid motion 15

4.4 Energy transport 15

4.5 Mass transport 16

5 Electrified methane pyrolysis process and techno-economic assessment 18

5.1 Process simulation 18

5.2 Techno-economic assessment 19

6 Supplementary Discussion 1: *The geometry of the wood carbon monolith* 20

7 Supplementary Discussion 2: Fibrous carbons formation 21

8 Supplementary Discussion 3: Rapid movement of fibrous carbon with H_2 flow 22

9 Supplementary Discussion 4: Spent wood carbon monoliths as promising SIB anode materials 22

Figures 24

Figure S1. Schematic diagram of a horizontal furnace system used for wood carbon monolithic reactor pyrolysis/carbonization. 24

Figure S2. Pictures of unmodified and modified spruce wood blocks and wood carbon monoliths. 25

Figure S3. SEM images of the wood carbon monolith along the radial (left) and the axial (right) directions. 26

Figure S4. Pore width distribution of the wood carbon monolith by applying CO_2 and N_2 adsorption and desorption test. 27

Figure S5. Schematic diagram of the direct induction heating system. 28

Figure S6. Effect of the temperature on CH_4 pyrolysis performance at a fixed GHSV of 750 h^{-1}. 29

Figure S7. Effect of the GHSV on CH_4 pyrolysis performance at a fixed temperature of 1150 ^oC. 30

Figure S8. Real-time detection data of the effluent gas via micro-GC a). before the wood carbon monolith being heated; b). during the methane pyrolysis test 31

Figure S9. Equilibrium converision of methane pyrolysis with temperature in this study using Aspen Plus v12. 32

Figure S10. a). Graphite crucible used in this study; b). SEM image of the carbon products obtained from CH_4 pyrolysis using the graphite crucible.. 33

Figure S11. SEM imgaes and the corresponding EDS elemental composition and distribution of fresh wood carbon monolith. 34

Figure S12. Raman spectra of fibrous carbon and spent carbon monolith. 35

Figure S13. Schematic diagram of the fibrous carbon formation and movement with the gas flow.. 36

Figure S14. Photos of carbon products : a).carbon deposited on the top of the wood carbon monolith; b). carbon deposited on the top cooling area of the connecting flanges; c). SEM image of the carbon products depositing on the top of the wood carbon monolith. 37

Figure S15. SEM images of channels (with certain carbon products leftover) in the spent wood carbon monolith. 38

Figure S16. a). Galvanostatic charge/discharge curves of half-call using fresh wood carbon monolith as anodes; b). Cyclic capability of fresh wood carbon monolith at a current density of 20 mA/g. 39

Figure S17. Cyclic capability of spent wood carbon monolith at a current density of 20 mA/g. 40

Figure S18. SEM image of fibrous carbon-filled channels in spent wood carbon monolith. 41

Figure S19. Schematic picture of the catalytic CH_4 dry reforming testing system. 42

Figure S20. Picture of metallic monolith reactor with enlarged view. 43

Figure S21. Equilibrium composition with temperatures of methane dry reforming in this study..44

Figure S22. XRD pattern of the Ni/MgO washcoat. 45

Figure S23. Temperature gradients in axial direction of different heating scenarios.......46

Figure S24. Space velocity distribution under distributed electrified heating within the reactor..47

Figure S25. Space velocity distribution under external heating within the reactor..........48

Figure S26. Obtained arrhenius plot for the CH_4 pyrolysis based on the experimental data. 49

Figure S27. Top and side view photos of the reactor during the reaction........................50

Figure S28. Effect of the amount of recycled tail gas to the specific energy demand of the production system...51

Figure S29. Breakdown of different costs of the distributed electrified heating-methane pyrolysis process (100kg/h natural gas, Natural gas price = 80 €/MWh, Electricity = 80 €/MWh). 52

Tables 53

Table S1. Specific surface areas and pore structure parameters for fresh and spent wood carbon monolith...53

Table S2. Flowrates used in this study and the corresponding space velocity.................54

Table S3. Comparison of the different technologies for methane pyrolysis for hydrogen and/or carbon production...55

Table S4. Ultimate and impurity analysis of the wood carbon monolith 56

Table S5. CH_4 pyrolysis kinetic parameters in present work 57

Table S6. Boundary conditions for the numerical model. 58

Table S7. Main process parameters and assumptions in Aspen Plus and calculations..... 59

Table S8. Summary of financial assumptions for economic analysis 60

Table S9. Input data for capital cost estimation.. 61

Table S10. Input data for operating cost estimation 63

Table S11. Summary of TEA on hydrogen production from methane from the literature64

Reference:... 66

1 Materials and Methods

1.1 CH_4 pyrolysis using wood carbon monolith reactor

1.1.1 Fabrication of the wood carbon monolith

The wood carbon monoliths used in this study were fabricated by pyrolysis and subsequent carbonization of a wooden cube with certain dimensions. Before the experiment, a Norwegian spruce strip of solid wood, which is often used in construction or civil engineering, was cut into a wooden cube with a length, width, and height of about 5 cm. For the wood carbon monoliths with modified geometry, extra holes with a diameter of around 1.9 mm were added into prepared wooden cubes by using an electrical drilling machine. As shown in ***figure S2***, 4*4=16 holes were drilled on each side of the wooden cube in total.

Before undergoing pyrolysis and carbonization, each wooden cube sample was weighed using a precise balance instrument. The sample was placed into a horizontal furnace, the schematic diagram of the horizontal furnace can be found in ***figure S1***. In order to eliminate all oxygen in the furnace, N_2 is injected into it at a flow rate of 200 L/min before heating. The sample was then heated at a low heating rate of 1 ^{o}C/min to a temperature of 800 ^{o}C, in order to prevent material expansion and cracking by the release of the volatiles. Once the set temperature was reached, the process was kept for 3 hours to eliminate all volatiles from the carbon monolith, which could affect the test performance during the CH_4 pyrolysis test. After the furnace cooled down, the produced wood carbon monolith was taken out and weighed again, yielding approximately 20% of its original mass.

1.1.2 CH_4 pyrolysis test

The overall system that was used for the CH_4 pyrolysis test is illustrated in ***figure S5***.

A PHILIPS ultra-high induction heating furnace is the major equipment involved, which has a maximum power of 10kW. It generates an induced magnetic field (from 30 to 500 kHz) through two turns of pure copper induction coils with currents up to 200A passing through. The copper coils have an outer diameter of 14 mm and an inner diameter of 10 mm. Moreover, its resistance is 2.066×10^{-3} Ω. In order

to maintain a safe working environment, water with a temperature below 5 ^oC is circulated through the copper coil to prevent it from overheating. To achieve this, a circulating condensate pump was utilized to keep the water cool and ensure it continuously flows through the copper rings. An additional system for CH_4 pyrolysis reactions was constructed based on the foundation of the induction furnace. This system consists of a gas supply system, mass flow control system, reaction tube equipped with a wood carbon monolith, and a gas product analysis instrument. To prepare for testing, the wood carbon monolith was placed into an aluminum insulation material with rounded exterior walls and a hollowed-out shape to fit the monolith for loading. The insulation material and carbon monolith are then inserted into a quartz tube with a diameter of 50 mm and a length of 500 mm. The outer wall of the insulation material is tightly fitted to the inner wall of the quartz tube to ensure complete gas passage through the wood carbon monolith. The quartz tube was placed vertically and connected to the gas supply and product gas analysis instrument through flanges at each end. The incoming CH_4 or Ar gas was regulated by mass flow controllers calibrated with an electrical soap film flowmeter. Gas analysis was performed using a gas chromatograph (μGC, 490 Micro-GC System QUAD, Agilent) with four columns - Molsieve 5 Å, PoraPLOT U, Al2O3/KCl, and CP-Sil 5CB. The gas volume was measured using a drum-type gas meter (TG1 type, Ritt Germany). An IR-thermometer (Optris CTlaser 3M) with an accuracy of ± 0.3% of reading +2 ^oC was also present at the top of the quartz tube to measure temperature in the range of 250 to 1800 ^oC.

Once the entire system was assembled, argon gas was injected (with a purity level greater than 99.99%) into the system at a 200 mL/min flow rate. Next, the induction furnace was started and the wood carbon monolith was heated up directly through eddy current. Once the temperature reached the necessary requirements, we switch the gas to pure CH_4 (with a purity level greater than 99.99%). The testing phase began with elevated temperatures of 850 ^oC, 950 ^oC, 1050 ^oC, and 1150 ^oC, at a fixed CH_4 flow rate of 100 mL/min (corresponding to a GHSV of 750 h^{-1}. The GHSV is computed by dividing the CH_4 flow rate by the volume of the carbon monolith. After it, the test was changed to the elevated flow rates of 100 ml/min (750 h^{-1}), 200 ml/min (1500 h^{-1}), 400 ml/min (3000 h^{-1}), and 800 ml/min (6000 h^{-1}), at a fixed temperature of 1150 ^oC by using a new wood carbon monolith. For each test condition, the reaction

persists for approximately 30 minutes. The stability of the test was also conducted at 1150 °C and 400 ml/min (3000 h^{-1}) by using a new wood carbon monolith. It had to be conducted in two rounds in accordance with laboratory safety management regulations. The first round lasted for 720 minutes, followed by cooling to room temperature under an argon atmosphere overnight. The second round of testing was initiated the next day after reheating and lasted for 480 minutes. After the test, solid carbons were collected from the wood carbon monolith's top surface and the connecting flanges' top cooling area. Certain carbons cannot be collected which means that the carbon yield is not able to be factored in. It is worth noting that a modified geometry wood carbon monolith has also undergone testing and has been found to perform identically to the unmodified wood carbon monolith.

1.1.3 CH_4 conversion, H_2 yield, and C_2H_2 yield calculation

The CH_4 conversion, H_2 yield, and C_2H_2 yield are calculated according to the following equations:

$$X_{CH_4} = \frac{F_{CH_4,0} - F_{CH_4}}{F_{CH_4,0}} \times 100\% \quad (1)$$

$$n = \frac{C_{C2H2}}{C_{H_2}} \quad (2)$$

$$Y_{H_2} = 2 \times (C_{CH_4,0} - C_{CH_4}) \times F_{CH_4,0} \times (1/(1+n)) \quad (3)$$

$$Y_{C2H2} = 2 \times (C_{CH_4,0} - C_{CH_4}) \times F_{CH_4,0} \times (n/(1+n)) \quad (4)$$

$$C_{H_2} = \frac{Y_{H_2}}{22.4 \times (Y_{H_2} + Y_{C2H2})} \quad (5)$$

where:

X_{CH_4}: CH_4 conversion, %

$C_{CH_4,0}$: CH_4 concentration at inlet i.e. 0.0445 mol/L (pure methane)

C_{CH_4}: CH_4 concentration, mol/L

n: molar ratio of C_2H_2 to H_2, calculated based on micro-GC

Y_{H_2}: hydrogen yield, mmol/min

Y_{C2H2}: C_2H_2 yield, mmol/min

$F_{CH4,0}$: methane inlet flow rate, mL/min

C_{H_2}: H_2 concentration, mol/L (excluding CH_4)

1.1.4 CH_4 pyrolysis over commercial graphite crucible

To study the impact of ash content on carbon product formation in CH_4 pyrolysis, the wood carbon monolith was substituted with a high-purity commercial hollow-bottom graphite crucible (99.99%, https://www.xwgraphite.com/pd.jsp?id=36#_jcp=3_25). All other test conditions were kept constant with the stability test. After the test, solid carbon products were collected from the interior of the graphite crucible.

1.2 Catalytic CH_4 dry reforming using a metal monolith reactor

1.2.1 Preparation of the metal monolith reactor with Ni/MgO catalyst washcoat

Metallic monolith without catalyst function was bought from Pingxiang Hualian Chemcial Ceramic Co. ltd (http://www.hlceramic.com/). Specially, the monolith, made of FeCrAl alloy, has a diameter of 35mm, cell density of 200 cpsi. The unit cell unit of the monolith is an equilateral triangle with a side length of about 0.3mm. Prior to catalyst coating, the metallic monolith was cut into a 32mm length using a water-cutting machine.

Coating of Ni/MgO catalyst on the metallic monolith is conducted by Hulteberg Chemistry & Engineering AB (https://www.hulteberg.com/), a company that specializes in providing catalyst coating services. The process started by cleaning the monolith to remove any impurities through calcination at 550 °C for 3 hours. Next, the monolith was dipped into a slurry containing MgO and a binder that attaches the MgO to the monolith surface. After that, the monolith was dried and calcinated at 850 °C for 3 hours under N_2 to remove the binder. This process was repeated until the monolith achieved the required amount of MgO. The MgO used in this study has a surface area of between 50 and 70 cm^2. The monolith was then dipped into Ni $(NO_3)_3$ solutions to impregnate Ni species on the surface of MgO. The monolith was then dried and calcinated at a temperature of 800°C for 3 hours under N_2 to obtain

NiO/MgO surface coating. This process was repeated until the required amount of Ni coating was achieved. In this study, the resulting coating was around 0.18 g NiO/1.89 g MgO on the metallic monolith with a volume of around 0.03 L, which equals a Ni loading of around 7 wt.%.

1.2.2 Catalytic CH_4 dry reforming test

The overall system that is used for the catalytic CH_4 dry reforming test is illustrated in ***figure S19***.

The reactor system consists of a horizontal tube (made from quartz tube) with the coated metallic monolith is placed in the middle of the tube. An induction heater is used to heat the metallic monolith. A diluted CH_4 gas cylinder with a CH_4 of 8% and N_2 as a gas balance is used in the experiment as the main reforming feedstock. CO_2 with purity of >99% is provided by another gas cylinder as a reforming agent. In addition, diluted H_2 gas cylinder with a H_2 of 4% and N_2 as a gas balance is used for the reduction of NiO/MgO catalyst into Ni/MgO. Prior to the test, the metallic monolith reactor was heated to a temperature of 500°C and a flow of 750 mL/min H_2/Ar for 8 hours to undergo reduction. The reactor is then heated to the specific testing temperature according to the test plan. After the temperature reaches the point, CH_4/N_2 mixture and CO_2 gases are injected into the metallic monolith reactor after passing through a gas mixture. During the test, the ratio of CH_4 to CO_2 is fixed at a value of 1, resulting in the composition of injecting gases of 92(N_2) :8(CH_4) :8(CO_2). The gas flow rate is set according to WHSV values used during the test. Specifically, for a WHSV of 75.8 L g_{cat}^{-1} h^{-1}, the flow rate of CH_4/N_2 mixture and CO_2 is set as around 2500 and 200 ml min^{-1}. Syngas producing from the metallic monolith reactor is then cooled and sent to the micro-GC for an online monitoring followed by a gas meter for the volume determination. Coke amount generated during the test is calculated according to the weight difference of the metallic monolith before and after test.

1.2.3 CH_4 and CO_2 conversion, syngas production capacity, H_2 and CO yield calculation

Potential reactions involved in the CH_4 dry reforming mainly include:

Dry reforming: $CH_4 + CO_2 = 2CO + 2H_2$ (6)

CH_4 cracking: $CH_4 = C + 2H_2$ (7)

Reverse water gas shift: $CO_2 + H_2 = CO + H_2O$ (8)

CO disproportionation: $2CO = CO_2 + C$ (9)

The CO yield is always higher than H_2 yield. This indicates the dominant side reaction ought to be the reverse water gas shift reaction. For ease of calculation, reverse water gas shift reaction is taken as the only side reaction. Therefore, in this study, CH_4 and CO_2 conversion, syngas production capacity, H_2 and CO yield are calculated according to the following equations:

$$X_{CH_4} = \frac{F_{CH_4,0} - F_{CH_4}}{F_{CH_4,0}} \times 100\% \quad (10)$$

$$X_{CO_2} = \frac{F_{CO_2,0} - F_{CO_2}}{F_{CO_2,0}} \times 100\% \quad (11)$$

$$Y_{H_2} = \left[2F_{CH_4} \times X_{CH_4} - \left(F_{CO_2} \times X_{CO_2} - F_{CH_4} \times X_{CH_4}\right)\right] / (1000 * 22.4 * 0.18) \quad (12)$$

$$Y_{CO} = \left[2F_{CH_4} \times X_{CH_4} + \left(F_{CO_2} \times X_{CO_2} - F_{CH_4} \times X_{CH_4}\right)\right] / (1000 * 22.4 * 0.18) \quad (13)$$

$$Y_{syngas} = Y_{H_2} + Y_{CO} \quad (14)$$

where:

X_{CH_4}: CH_4 conversion, %

$F_{CH_4,0}$: CH_4 flow rate at inlet, ml min^{-1}

F_{CH_4}: CH_4 flow rate at outlet, ml min^{-1}

X_{CO_2}: CO_2 conversion, %

$F_{CO_2,0}$: CO_2 flow rate at inlet, ml min^{-1}

F_{CO_2}: CO_2 flow rate at outlet, ml min^{-1}

Y_{syngas}: syngas production capacity of the metallic monolith, L h^{-1}

Y_{H_2}: H_2 yield, mol/ g_{Ni}^{-1} h^{-1}

Y_{co}: CO yield, mol/ g_{Ni}^{-1} h^{-1}

2 Sample characterization

Texture properties of the fresh and spent wood carbon monolith are determined by obtaining N_2 and CO_2 adsorption–desorption isotherms obtained at 77 K and 298 K by using a Micromeritics model

ASAP 2020 instrument. Applying the Brunauer-Emmett-Teller (BET) equation to the N2 adsorption isotherm calculates the sample's surface area. Pore size analysis based on N_2 and CO_2 adsorption–desorption isotherms is performed by applying density functional theory calculations.

Scanning Electron Microscope (SEM) observation is carried out by using a JEOL JSM-7800F instrument (20 kV and 10 mm working distance) instrument equipped with a Bruker AXS XFlash Detector 4010 (MA, USA). Energy Dispersive Spectroscopy (EDS) detector from Oxford Instrument is further equipped on the SEM instrument to perform the samples' elemental composition and surface mapping analysis.

TEM was performed at room temperature on a JEOL JEM-2100 microscope equipped with a LaB6 gun operated at 200 kV. Samples dispersions in ethanol were dropped on Cu TEM grids with holey carbon films and dried in air. TEM observation and image acquisition were performed utilizing a Gatan SC1000 ORIUS CCD camera.

Ultimate elemental analysis and the corresponding ash content and composition analysis of the wood carbon monolith sample were conducted by Eurofins Biofuel & Energy Testing Sweden AB (*https://www.eurofins.com/*).

Raman spectra were obtained by using a Tyrode I Raman microscope equipped with a 532-nm wavelength diode laser. The power of the laser is 50 mW, and the accumulated time is 90 s.

3 Electrochemical performance test

In order to evaluate the effectiveness of spent wood carbon monolith as anodes for sodium-ion batteries, pouch-type half-cell batteries are assembled and tested. The carbon monolith is ground and sifted, using only samples with a size of <32 μm. In order to make a working electrode, spent carbon monolith material and CMC (sodium salt of carboxymethyl cellulose) is mixed in a weight ratio of 95:5. Thereafter, they are blended together in water using a pestle mortar, where per gram of total mixed sample, 3 mL of DI water was used. The prepared slurry is coated on a C-coated Al foil (using 350 μm doctor’s blade) and dried at ambient temperature. Then it is punched into circular disks (diameter = 13

mm) to make the electrodes. The electrodes are then moved into a Buchi oven for drying at 120 °C overnight. The Buchi oven is placed inside an MBraun Labstar glovebox filled with argon (H_2O/O_2 level ≥ 0.5 ppm). Na disk with a height of 15 mm is prepared and used as the counter electrode. Two sheets of solution with 80 µL of 1 M NaPF6/Diglyme are used as the electrolyte. To evaluate the electrochemical performance, the cell is subjected to galvanostatic cycling using a LAND potentiostat instrument. This is carried out in CC mode, at a current of 20 mA/g, within the voltage range of 0.001 to 2.5 V, and at a temperature of 25 °C.

4 Computational fluid dynamic (CFD) simulations

The model was implemented in COMSOL Multiphysics 6.0 in a 2D-axisymmetric geometry with fully coupled equations for fluid motion, energy transport, and mass transport. The simulation uses CH_4 as the reactant gas for pyrolysis reactions.

4.1 Geometry and heating scenarios definition

The reactor model is simplified by treating it as a porous medium with pore size and porosity similar to the wood carbon monolith since simulating numerous hierarchical, elongated, and tortuous micron-scale channels is challenging. Wood carbon monolith with unmodified geometry is used. Specifically, the used geometry structure of the numerical model is a square with a side length of 0.02 m. The porous media is modeled using an equivalent porous media model, of which the void ratio and average pore diameter are 0.82 and 0.06 mm, respectively.

External and induction heating are simulated as boundary and domain heating of the porous media, respectively. The simulation of electromagnetic induction heating is simplified by setting the solid temperature at a constant value that is achieved by the experiments. Heat is derived from the boundary with a fixed temperature for boundary heating, and heat is derived from the solid porous medium (skeleton) with a fixed temperature for domain heating. Both are consistent with the characteristics of the corresponding heating method. The energy transfer efficiency of both heating methods, especially induction heating, is not taken into account. The primary focus of this research is to examine the

variations in gas reactant heating caused by two heating methods. Induction heating is not the sole method for achieving uniform heating of the wood carbon monolith.

4.2 Boundary equations

Table S6 lists the boundary equations for the numerical models with boundary heating and domain heating, respectively.

4.3 Fluid motion

The gas mass conversion equation is described by:

$$\nabla \cdot (\rho_g \mathbf{u}_g) = R_{CH_4} + R_{H_2} \tag{15}$$

where ρ_g, and $\mathbf{u}_g$ are the gas density and gas velocity.

$\mathbf{u}_g$ is calculated by Darcy's Law:

$$\mathbf{u}_g = -(\kappa_p/\mu_g)\nabla p_g \tag{16}$$

where κ_p, μ_g, and p_g are the porous media permeability, gas dynamic viscosity, and gas pressure.

The gas transport equation is determined by:

$$\mathbf{u}_g \cdot \nabla(c_i) = \nabla \cdot (\varepsilon_p D_i \nabla c_i) + R_i \tag{17}$$

where ε_p is the porous media porosity, i represents gas species (methane and hydrogen), and D_i is the diffusion coefficient.

4.4 Energy transport

This study uses local thermal non-equilibrium (LTNE) to calculate solid and gas temperatures. Therefore, the solid and gas energy conversion equations are determined by Eqs. (18) and (19):

$$0 = \nabla \cdot (k_{s,eff} \nabla T_s) + h_{sg}(A_{s,sp}/V_{sp})(T_g - T_s) \tag{18}$$

where T_s, $k_{s,eff}$, h_{sg}, $A_{s,sp}/V_{sp}$, and T_g are the solid (porous media) temperature, solid effective thermal conductivity, interfacial heat transfer coefficient, surficial area per unit volume and gas temperature.

$$\nabla \cdot (\rho_g C_{p,g} \mathbf{u}_g T_g) = \nabla \cdot (k_{g,eff} \nabla T_g) + h_{sg}(A_{s,sp}/V_{sp})(T_s - T_g) - R_{CH_4} \Delta H_{CH_4} \tag{19}$$

where $C_{p,g}$, $k_{g,eff}$, and ΔH_{CH_4} are the gas heat capacity, gas effective thermal conductivity, and reaction enthalpy.

Heat transfer in solid takes into account radiation and is calculated using the Rosseland approximation. Therefore, $k_{s,eff}$ and $k_{g,eff}$ are calculated by:

$$k_{s,eff} = \frac{1-\varepsilon_p}{3} k_s + 16\sigma d_p T_s^3/3 \quad (20)$$

$$k_{g,eff} = \varepsilon_p k_g \quad (21)$$

where k_s, σ, d_p, and k_g are solid thermal conductivity, Stefan–Boltzmann constant, pore diameter, and gas thermal conductivity.

h_{sg} and $A_{s,sp}/V_{sp}$ are calculated by:

$$h_{sg} = \frac{k_g(2+1.1Pr^{1/3}Re^{0.6})}{d_p} \quad (22)$$

$$\frac{A_{s,sp}}{V_{sp}} = \frac{6(1-\varepsilon_p)}{d_p} \quad (23)$$

where Pr and Re are the Prandtl number and Reynolds number of the fluid.

4.5 Mass transport

The CH_4 pyrolysis reaction is described by [1]:

$$CH_4 \leftrightarrow C_s + 2H_2, \Delta H^0_{25°C} = 74.8 kJ/mol \quad (24)$$

The CH_4 pyrolysis is calculated by applying Eq. (24) using the calculated E_a and k_0 from the kinetic study:

$$r_{CH_4} = c_{CH_4} \cdot 1.6 * 10\hat{\ }5 \cdot \exp\left(-\frac{91010}{RT}\right) \quad (25)$$

Consequently, the CH_4, carbon, and H_2 reactions rates are determined by:

$$R_{CH_4} = -r_{CH_4} \quad (26)$$

$$R_{C_s} = r_{CH_4} \quad (27)$$

$$R_{H_2} = 2r_{CH_4} \tag{28}$$

Kinetic study

The kinetic model is calculated based on the experimental data using following equations.

A first-order rate expression for CH_4 pyrolysis was used to mode kinetics:

$$-r_{CH4} = kC_{CH4} \tag{29}$$

$$\mathrm{k} = \frac{X(1+\varepsilon X)}{(1-X)\,\tau} \tag{30}$$

where:

$-r_{CH4}$: rate of reaction, mol • L^{-1}·s^{-1}

k: rate constant, s^{-1}

C_{CH4} : CH_4 concentration, mol·L^{-1}

τ : mean residence time, s

X: chemical conversion, dimensionless

ε: volume expansion factor, assumed 0.5 in the present study

The temperature dependence of k is determined by imposing an Arrhenius’ law:

$$\mathrm{k(T)} = k_0 \exp\left(\frac{-E_a}{RT}\right) \tag{31}$$

where:

Ea: activation energy, kJ/mol

k0: frequency factor, units of the corresponding rate constant

T: absolute temperature, K

R: gas constant (8.314 J/mol/K)

Figure S26 shows the Arrhenius plot for determination of apparent activation energy and frequency factor of the present CH_4 pyrolysis reaction. E_a and k_0 are determined by linear regression and results are listed in ***table S5***.

5 Electrified methane pyrolysis process and techno-economic assessment

5.1 Process simulation

A system for co-producing hydrogen and carbon is suggested based on a distributed electrified methane pyrolysis reactor that has been investigated. The proposed system, illustrated in **Fig. 6A**, comprises a primary pyrolysis reactor heated through induction, a cyclone for separating the carbon product, a heat recovery system, a multi-stage compressor, and a pressure swing adsorption (PSA) unit. The primary feedstock used was natural gas with a composition typical of that obtained from a reference [2] (***table S7***). The process model was created using ASPEN PLUS V12.1 (Aspen Technology, Inc) to conduct heat and mass balance calculations, taking into account thermodynamic equilibrium for the methane pyrolysis process. The selected equation of state was the Soave–Redlich–Kwong (SRK) model. This model asserts that all thermodynamic properties, including chemical potential, are determined based on the relationship between pressure, volume, and temperature of pure components and mixtures [3]. The pyrolysis reactor was operated at 1150 °C, assuming complete conversion of the hydrocarbon gases. The reactor was simulated using the RGIBBS reactor module, which utilizes the Gibbs free energy minimization method to estimate product composition. The PSA model was simplified by incorporating a separator block with a 90% H_2 recovery, representative of the industrial standard [4]. To optimize H_2 and carbon production at a minimum specific electricity consumption (kWh/Nm^3-H_2), a portion of the tail gas discharged from the PSA is recirculated to the pyrolysis reactor. Through sensitivity analysis, it was determined that the minimum specific electricity consumption is attained with a 70% tail-gas recycle. The main process parameters and asscumptions in Aspen Plus and calculations are summarized in ***table S7***.

5.2 Techno-economic assessment

The key economic indicator employed is the Levelized Cost of Hydrogen (LCOH), which is a measure of the average cost to produce a unit of hydrogen over the lifetime of a project or facility. It is estimated using the total discounted costs over the total discounted hydrogen production, as presented using formula below:

$$LCOH= \frac{\sum_{t=1}^{n}\frac{C_t+O_t-R_t}{(1+r)^t}}{\sum_{t=1}^{n}\frac{H_t}{(1+r)^t}} \qquad (32)$$

where

It : Capital investment in year t, €

Ot : Operational expense in year t, €

Rt : Revenue generated from the sale of co-products in year t, €

Ht : Hydrogen output in year t, kg

r: Interest rate, %

t: each year of operation

n: plant lifetime, years

Table S8 presents the main assumptions applied in the economic assessment.

Capital Expense Estimation

The estimation of equipment purchases prices, crucial for determining capital costs, is derived using general cost correlations for standard items like heat exchangers and cyclone as per [5] and employs simplified scaling correlations for more complex units (see Eq. 33), with cost information sourced from existing literature. The mass balance derived from the Aspen simulation results is used for sizing the equipment. A scaling function is applied to account for the effect of scale on unit costs:

$$\frac{Cost_a}{Cost_b} = \left(\frac{Size_a}{Size_b}\right)^R \qquad (33)$$

where R is the scaling factor.

The present study scaled the cost of the pyrolyser based the costs of small-scale gas-fired furnace [6] and inductive electrical heating elements [7], while the cost of PSA is taken from [8]. The detailed breakdown of costs and scaling factors for each process unit is available in ***table S9***. To update these

estimates to reference year of 2022 values, they were adjusted for inflation using the Chemical Engineering Plant Cost Index (CEPCI), as detailed in Equation (34).

$$\text{Cost}_{\text{€ Year Y}} = \text{Cost}_{\text{€ Year x}} \cdot \frac{\text{CEPCI}_{\text{Year Y}}}{\text{CEPCI}_{\text{Year X}}} \quad (34)$$

Estimates for Total Capital Investment (TCI) are derived from the purchase costs of equipment, adjusted by using scaling factors provided [9] specific to solid/fluid processing plants (F = 4.87). Additionally, a 20% contingency is included considering the substantial uncertainty involved in the cost evaluation of the inductive-heated pyrolyzer unit. This method of estimation is expected to have an accuracy of around ± 30%. The specific factors employed in the calculation of TCI can be found in ***table S9***.

Operating Expense Estimation

The operating costs encompass several components: (i) the expenses associated with feedstock and utilities, (ii) credits from additional carbon products, (iii) variable operational expenses as detailed in ***table S10***, and (iv) revenue from the export of district heating.

6 Supplementary Discussion 1: *The geometry of the wood carbon monolith*

Natural spruce wood with dimensions of approximately 4 cm * 4 cm * 4 cm is used for the laboratory-scale 3D wood carbon monolithic reactor fabrication (***figure S2***). After a pyrolysis and carbonization process at 800 ºC for 3 hours (refer to ***figure S1*** for the furnace used), a 3D carbon monolith with dimensions approximately 2 cm * 2 cm * 2 cm was obtained (***figure S2***). Hierarchical, open, elongated and tortuous channels with diameters of 10-60 μm in the axial direction were observed from the scanning electron microscopy (SEM) images of the 3D wood carbon monolith (***figure S3***). According to reports, these channels enable efficient gas flow while maintaining reasonable pressure drops [10, 11]. Nitrogen adsorption and desorption test proved the presence of secondary mesopores (2-25 nm) and micropores (0.5-2 nm) on the channel walls, which potentially further facilitate the mixing and diffusion of gases between different channels (***figure S4** and **table S1***). The tortuous channels combined with secondary mesopores and micropores can help to increase the gas residence time inside the monolith during the test [10, 12], which is favourable for CH_4 decomposition. Moreover, the geometry of the carbon

monolith is easy to modify. This work also conducts an example of manually drilling additional holes to include new gas channels (***figure S2***).

7 Supplementary Discussion 2: Fibrous carbons formation

Distributed electrified heating produces solid carbons in the form of curled fibrous carbons (**Fig. 2C**), as opposed to the typical spherical carbon blacks produced through direct CH_4 pyrolysis without a metal catalyst [13]. These fibrous carbons have diameters that fall within two size ranges: 10-100 nm and 300-1000 nm (**Fig. 2C**). Upon examining the transmission electron microscopy (TEM) images, it is apparent that fibrous carbons show a combination of constructions, some with hollows resembling bamboo-like nanotubes and some without hollows resembling nanofibers (**Fig. 2C**). Carbon nanofibers and carbon nanotubes, which have greater commercial value than carbon blacks, are commonly formed with the aid of a metal catalyst [14]. The extensive production of carbon nanofibers and carbon nanotubes by using a metal-free wood carbon monolith and the high purity of the carbon products (no risk of metal contamination) indicate other disruptive advantages of our technology (***table S3****)*. We also conduct CH_4 pyrolysis using an induction-heated graphite crucible with a high purity of 99.99%. The carbon products obtained are in the form of fibers (***figure S10***), excluding the effect of the mineral impurities in the wood carbon monolith (<1 wt. %, ***figure S11***, and ***table S4***) on the formation of the fibrous carbons. It has been reported that carbon nanofibers and nanotubes can be synthesized through microwave-assisted pyrolysis of CH_4 using high-purity activated carbons [15, 16] due to its "hot spots" effect, where the microwave energy is more concentrated. Induction heating has the similar features with the microwave heating, which allows for concentrated localized heating of the samples by inducing eddy currents. The fibrous carbon products collected in this study have a relatively low graphitization degree (***figure S12***), which is consistent with the carbon products produced from microwave-assisted processes [16]. As a result, it can be inferred the concentrated localized heating effect of the induction heating facilitates the accumulation of carbon atoms into fibers (***figure S13***). Additionally, the newly produced carbons within the channels possess the ability to be induction-heated, showcasing the autocatalytic impact of the carbons observed in the microwave-assisted procedure [17]. This may enhance the buildup of carbon atoms into fibers as well (***figure S13***).

8 Supplementary Discussion 3: Rapid movement of fibrous carbon with H_2 flow

One of the biggest obstacles in implementing this technology is the risk of micron-scale carbon channel blockages caused by fibrous carbons. These blockages can cause the reaction to shut down. However, it is confirmed that solid carbons can be quickly carried out from the carbon channels during testing, ensuring a continuous process described previously. Most of the fibrous carbons are located on the top of the carbon monolith (free falling by gravity) and in the top cooling area of the connecting flanges after the test (***figure S14***). The channel size, which ranges from 10-60 μm, is notably larger than the bulk size of the fibrous carbon, which is less than 1 μm. As a result, most solid carbons are carried with the gas flow, with only a small portion attaching to the walls of the channel (***figure S1***5). When comparing the fresh carbon monolith to the spent one, the reduction in specific surface area is a clear indication of secondary pores (on channel walls) closure by deposition of carbon (***table S1***). The leading cause of deactivation in activated carbon catalysts for CH_4 pyrolysis has been reported as the closure of the inner pores within the same range as the secondary pores on the channel wall [18, 19]. In this study, the closure of these pores on the channel does not appear to result in a decrease in the CH_4 conversion rate, as the main channels remain unimpeded (***figure S15***).

9 Supplementary Discussion 4: Spent wood carbon monoliths as promising SIB anode materials

Numerous potential uses for high-purity fibrous carbons have been thoroughly researched. The performance of our fibrous carbon products in specific applications will be assessed in the future. The only carbon product requiring effective utilization is the spent wood carbon monolith. Even though the wood carbon monolithic reactors can function reliably for a certain period, they will eventually reach the end of their life cycle. The disposal of these spent wood carbon monoliths will need to be considered. Heat treatment of amorphous wood carbon induces the formation of anisotropic graphite microcrystals (known as hard carbon, **Fig. 2E**), making it an efficient anode material for sodium-ion batteries (SIBs). Moreover, it has been reported that depositing extra soft carbon onto the porous surface of hard carbon can effectively close the open pores in hard carbon, resulting in a significant increase in the initial

coulombic efficiency (ICE) for SIBs [20]. The reduction in the specific surface area of the spent wood carbon monolith clearly indicated the nanopores closure by deposition of fibrous carbons, which is a kind of soft carbon (***table S1***). ***Figure S18*** *shows* certain channels filled by deposited fibrous carbons. As part of our research, an electrochemical study is conducted to assess the effectiveness of utilizing certain spent wood carbon monoliths (specifically, those that had undergone a 1200-minute stability test) as anodes for SIBs. The prepared SIB half-cell demonstrated a remarkable ICE value of 94.7% and maintained a decent revisable capacity of 218.9 mAh/g (**Fig. 2D**, ***figure S17***). These values are much higher than that demonstrated by the fresh carbon monolith (**Fig. 2D**, ***figure S16***). The stability of the electrode material was evident from the 20-cycle charge-discharge curve (**Fig. 2E**, ***figure S17***). These findings suggested that the spent wood carbon monolith could be a viable option for SIB anode materials with remarkable ICE value.

Figures

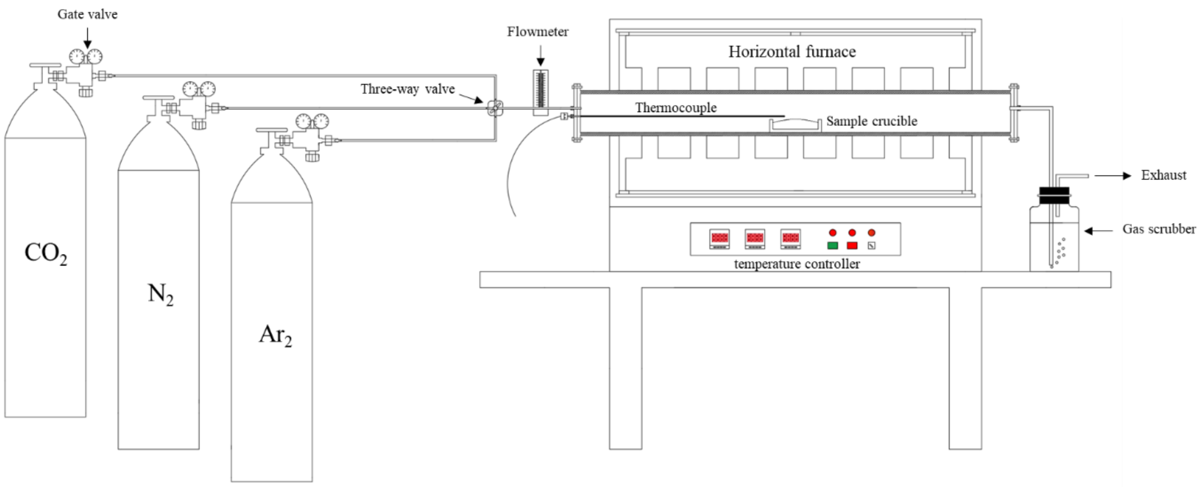

Figure S1. Schematic diagram of a horizontal furnace system used for wood carbon monolithic reactor pyrolysis/carbonization.

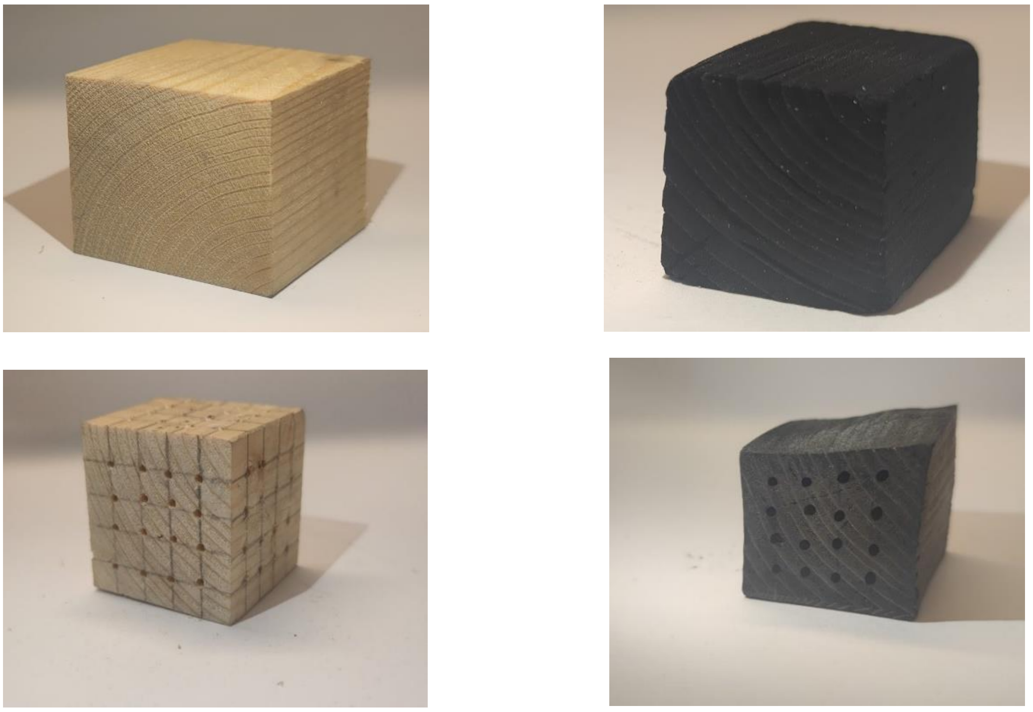

Figure S2. Pictures of unmodified and modified spruce wood blocks and wood carbon monoliths.

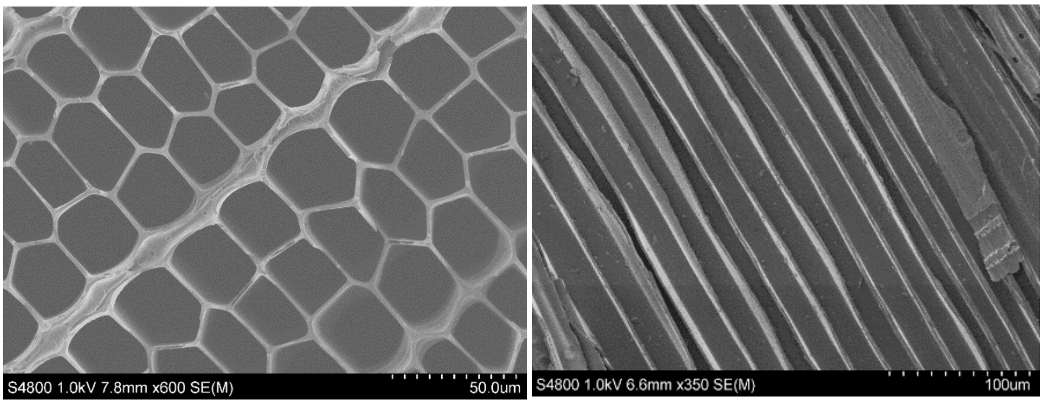

Figure S3. SEM images of the wood carbon monolith along the radial (left) and the axial (right) directions.

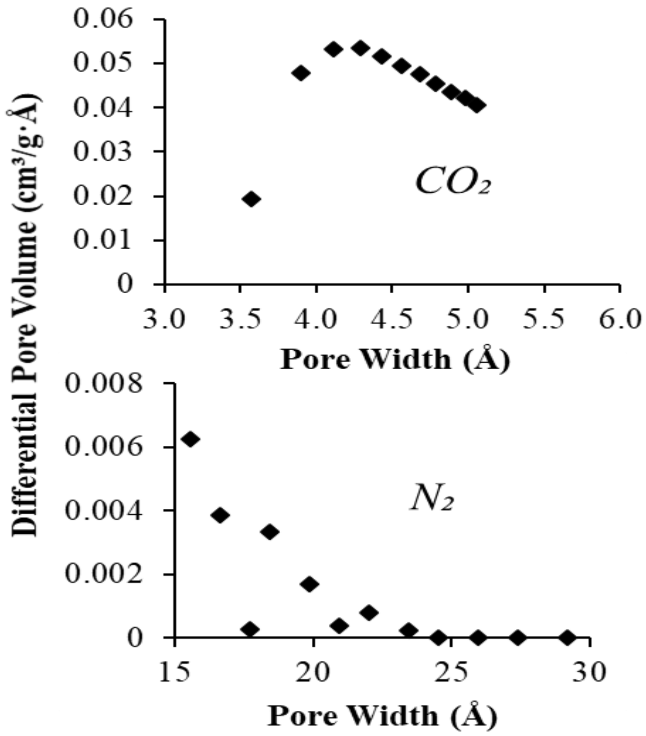

Figure S4. Pore width distribution of the wood carbon monolith by applying CO_2 and N_2 adsorption and desorption test.

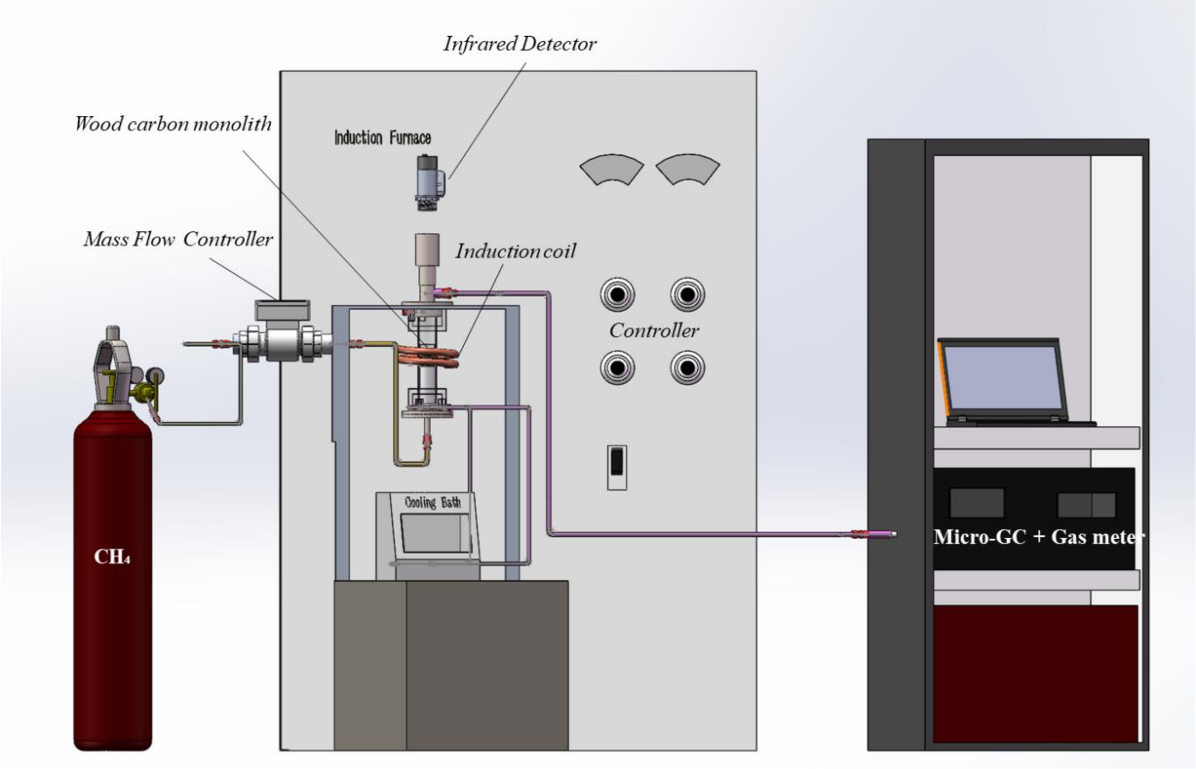

Figure S5. Schematic diagram of the direct induction heating system.

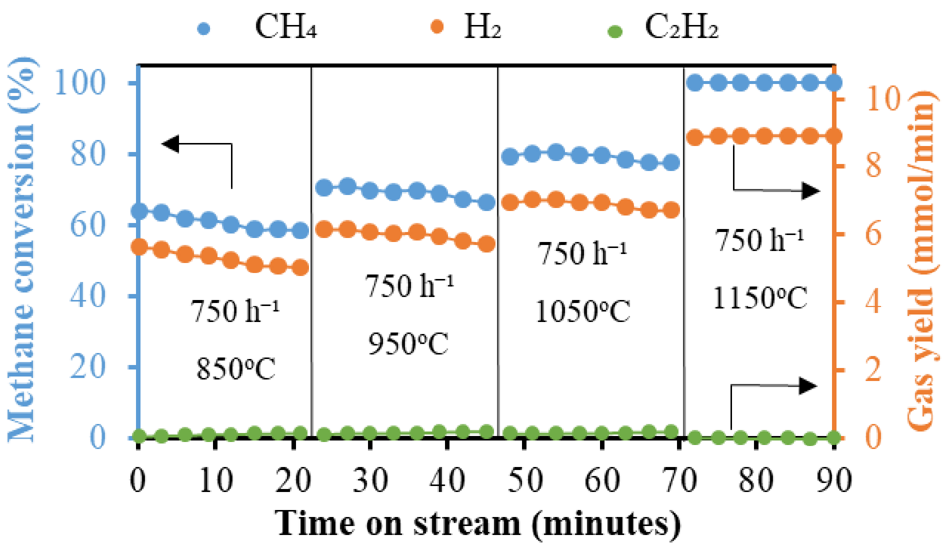

Figure S6. Effect of the temperature on CH_4 pyrolysis performance at a fixed GHSV of 750 h^{-1}.

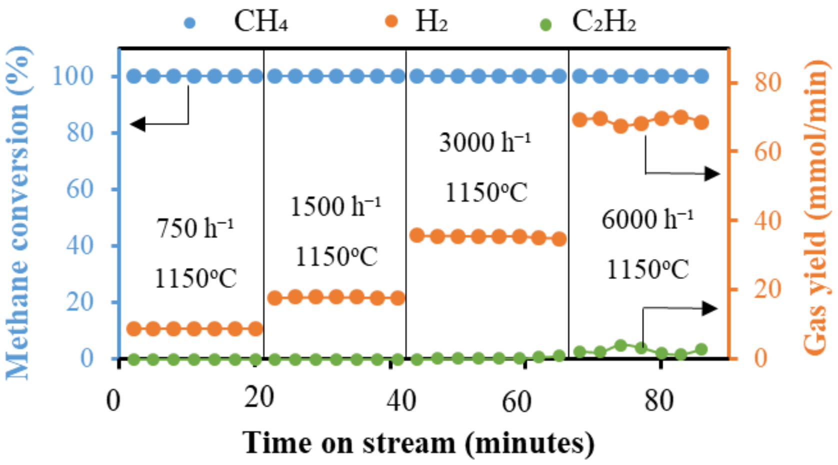

Figure S7. Effect of the GHSV on CH_4 pyrolysis performance at a fixed temperature of 1150 ºC.

a) Before

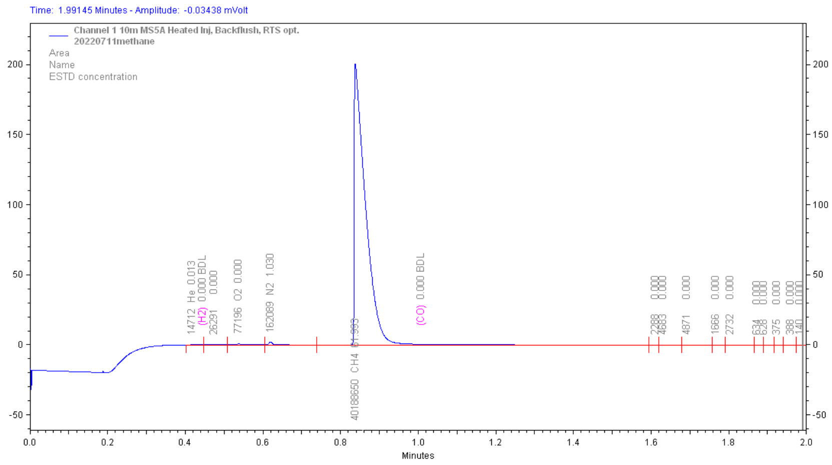

b) During test

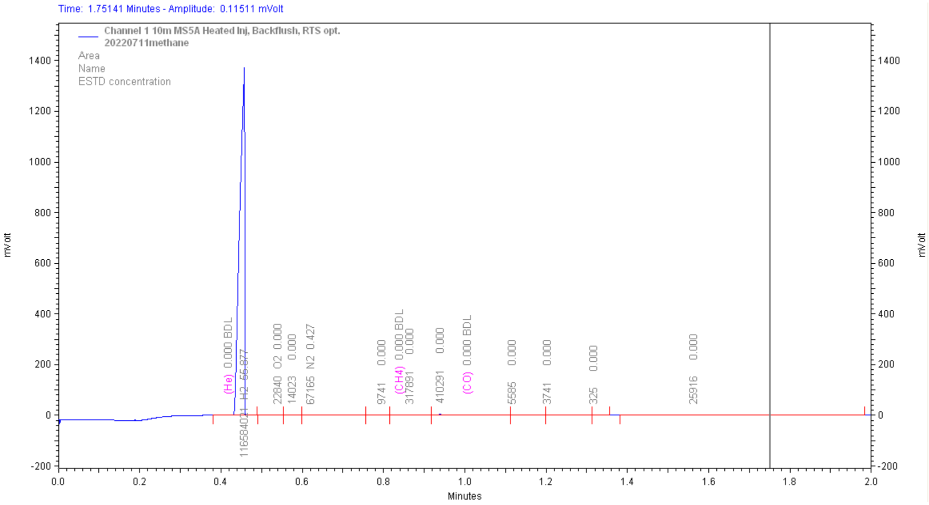

Figure S8. Real-time detection data of the effluent gas via micro-GC a). before the wood carbon monolith being heated; b). during the methane pyrolysis test

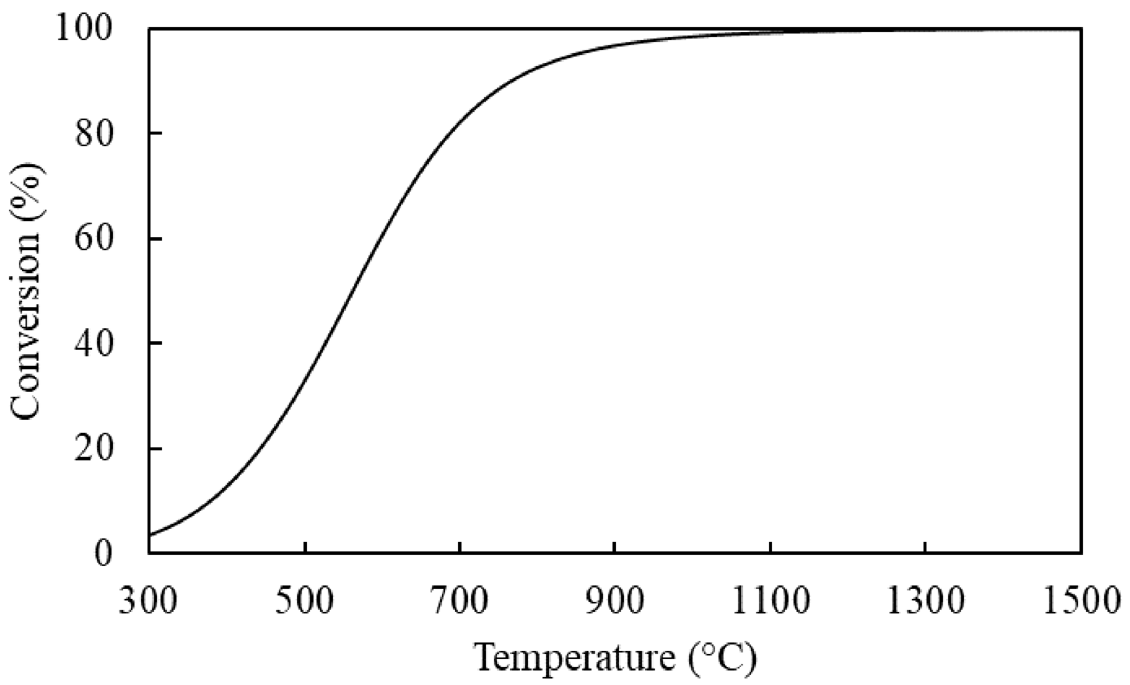

Figure S9. Equilibrium converision of methane pyrolysis with temperature in this study using Aspen Plus V12.

a)

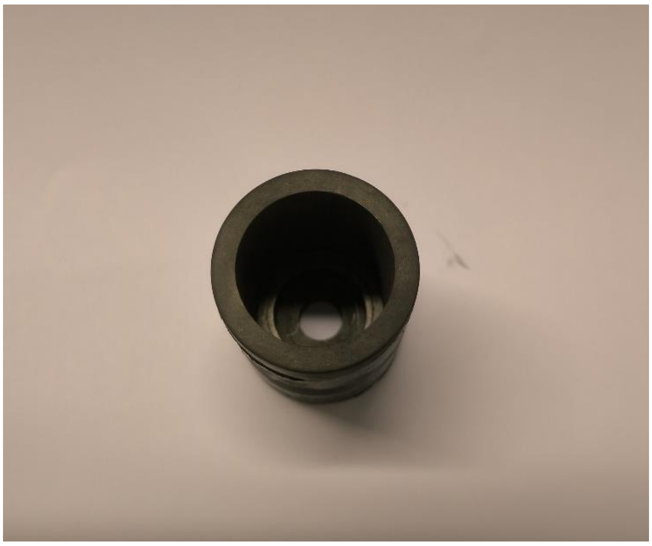

b)

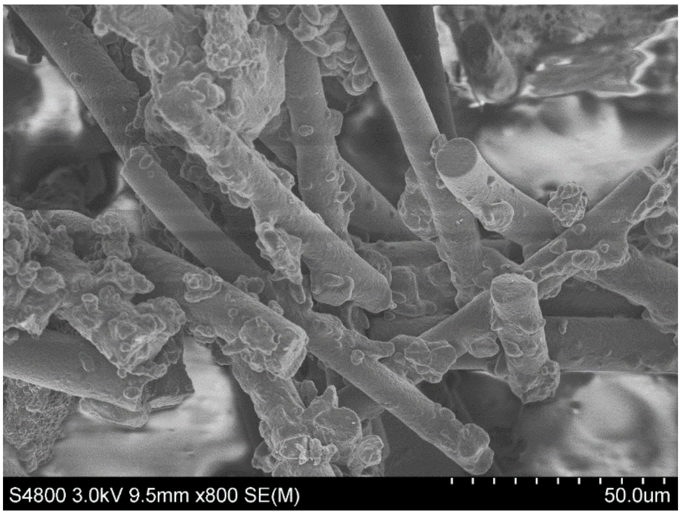

Figure S10. a). Graphite crucible used in this study; b). SEM image of the carbon products obtained from CH_4 pyrolysis using the graphite crucible.

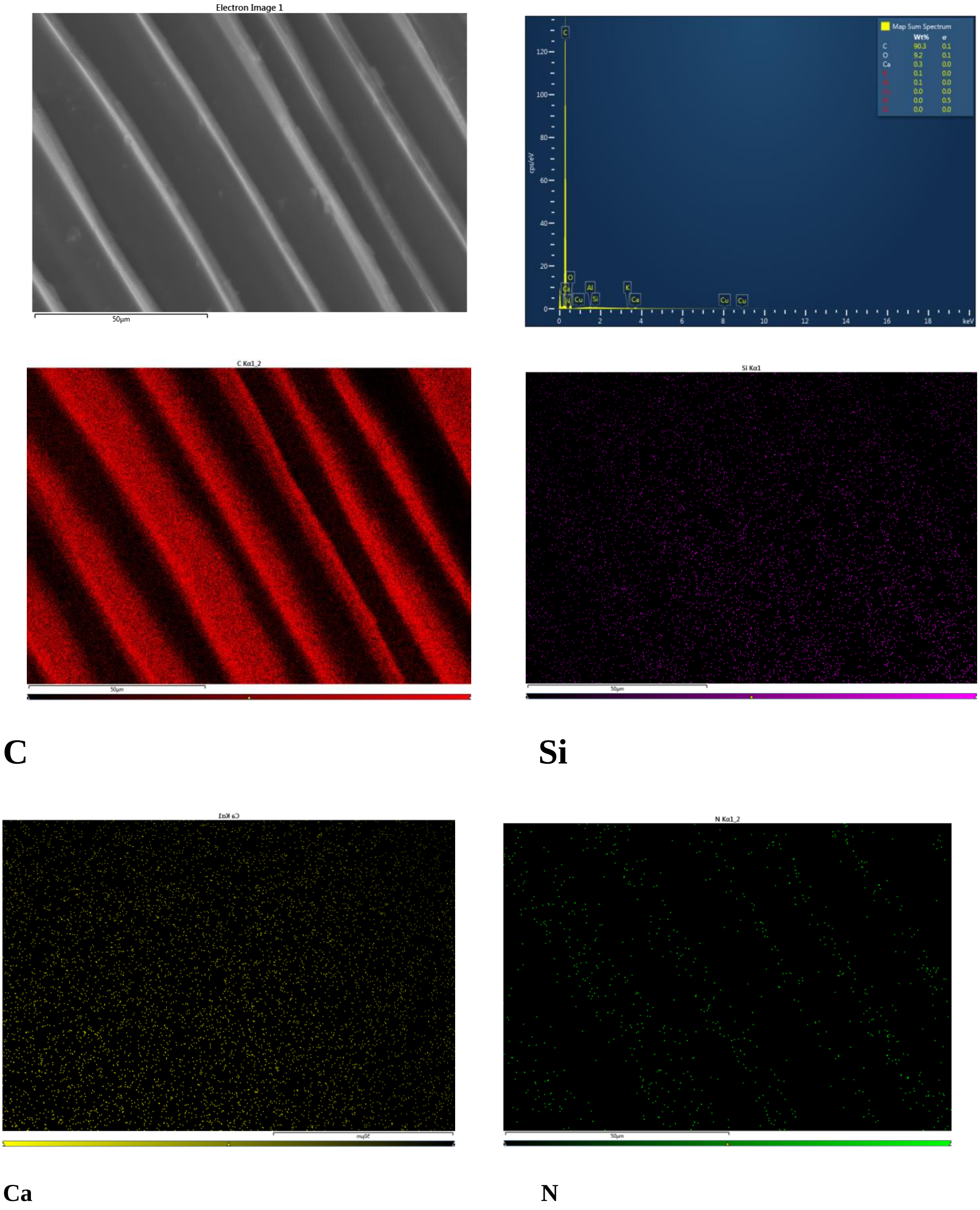

Figure S11. SEM imgaes and the corresponding EDS elemental composition and distribution of fresh wood carbon monolith.

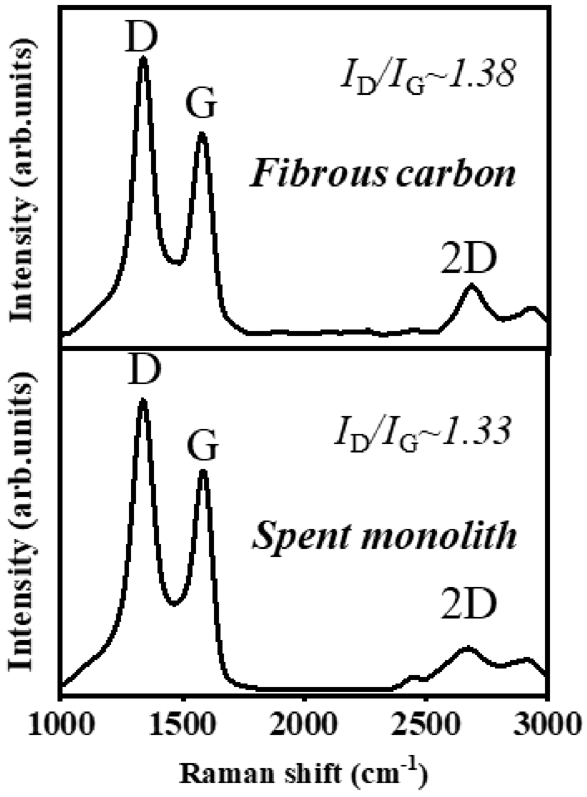

Figure S12. Raman spectra of fibrous carbon and spent carbon monolith.

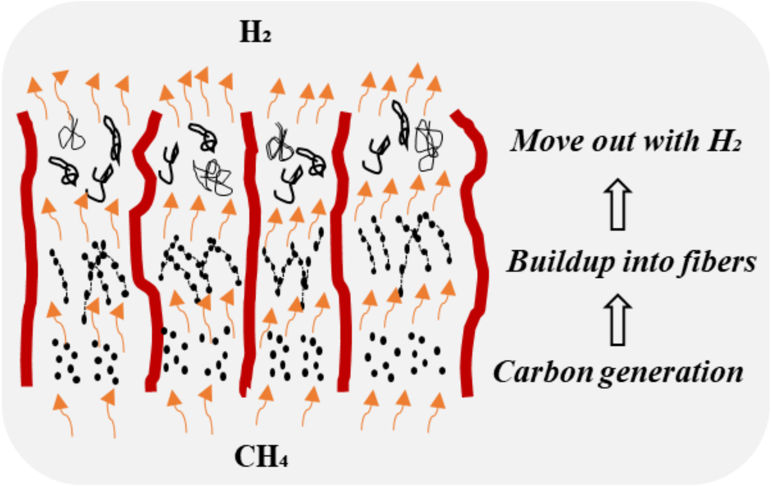

Figure S13. Schematic diagram of the fibrous carbon formation and movement with the gas flow.

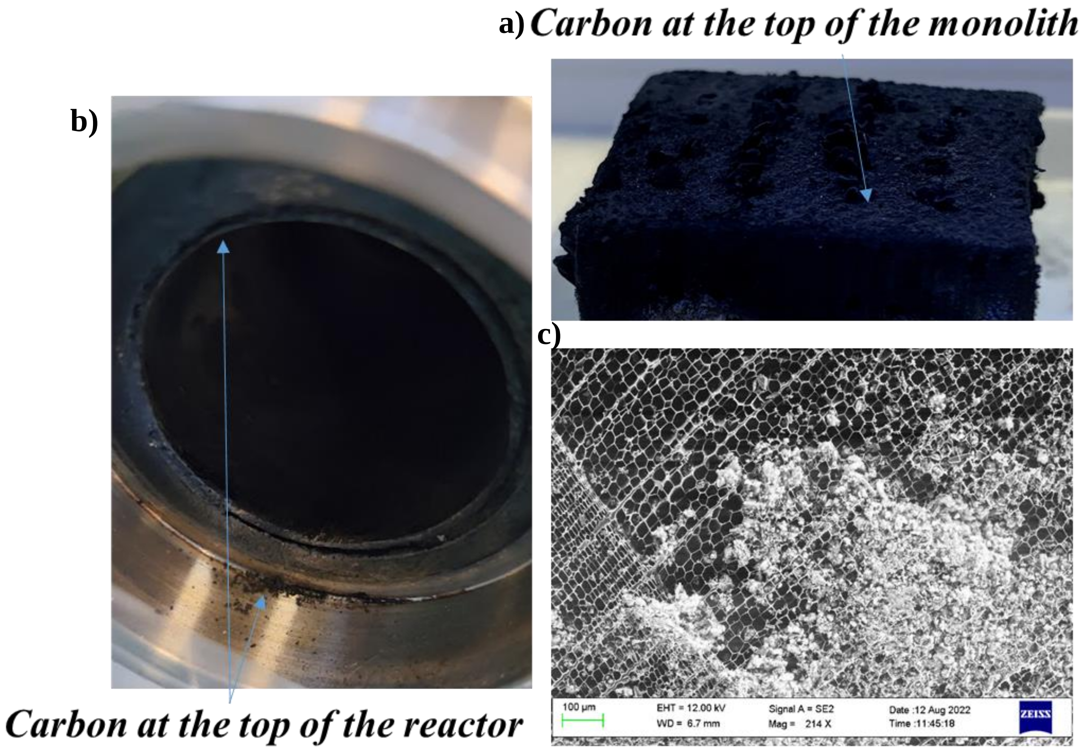

Figure S14. Photos of carbon products : a).carbon deposited on the top of the wood carbon monolith; b). carbon deposited on the top cooling area of the connecting flanges; c). SEM image of the carbon products depositing on the top of the wood carbon monolith.

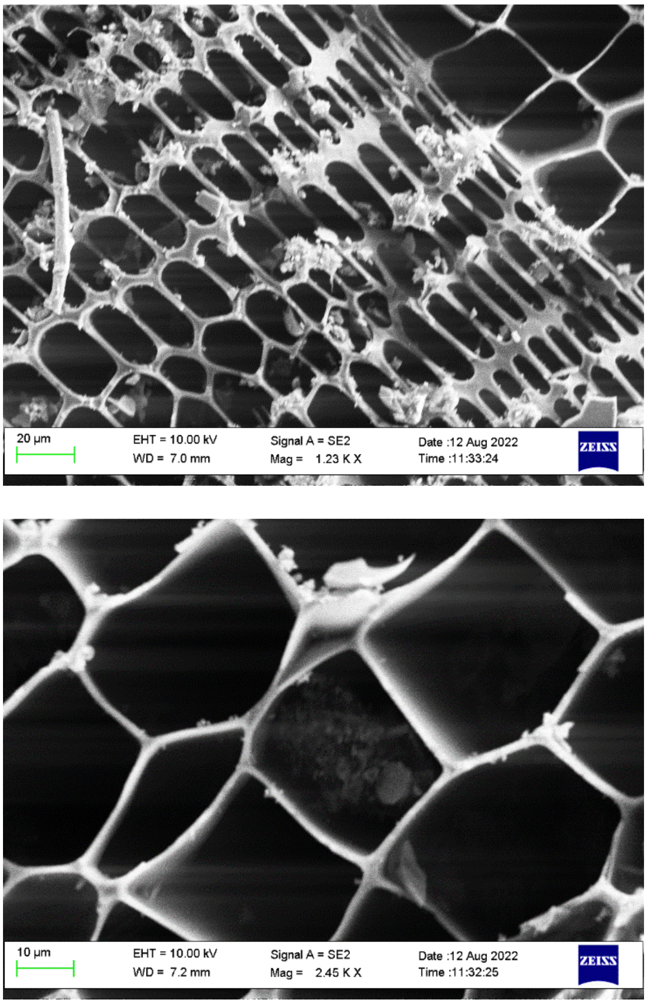

Figure S15. SEM images of channels (with certain carbon products leftover) in the spent wood carbon monolith.

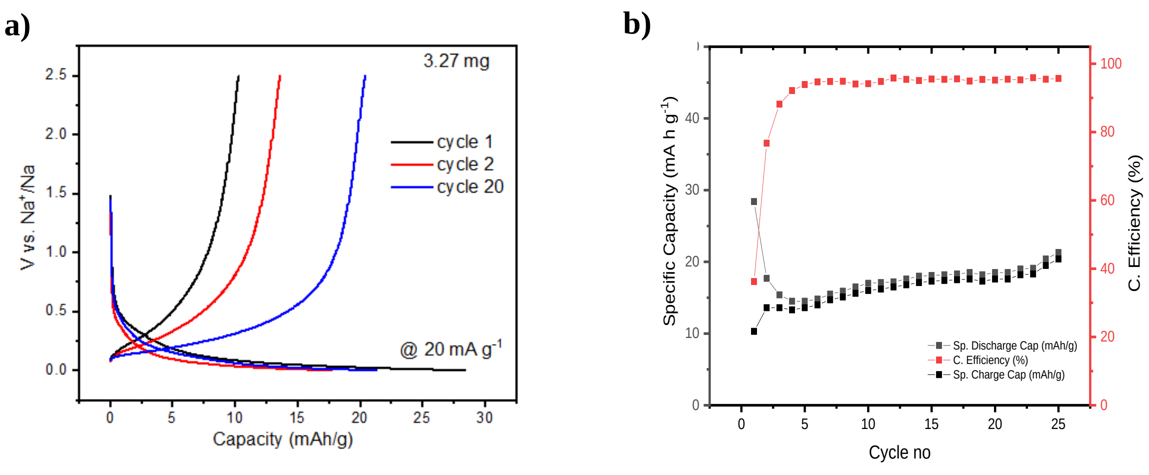

Figure S16. a). Galvanostatic charge/discharge curves of half-cell using fresh wood carbon monolith as anodes; b). Cyclic capability of fresh wood carbon monolith at a current density of 20 mA/g.

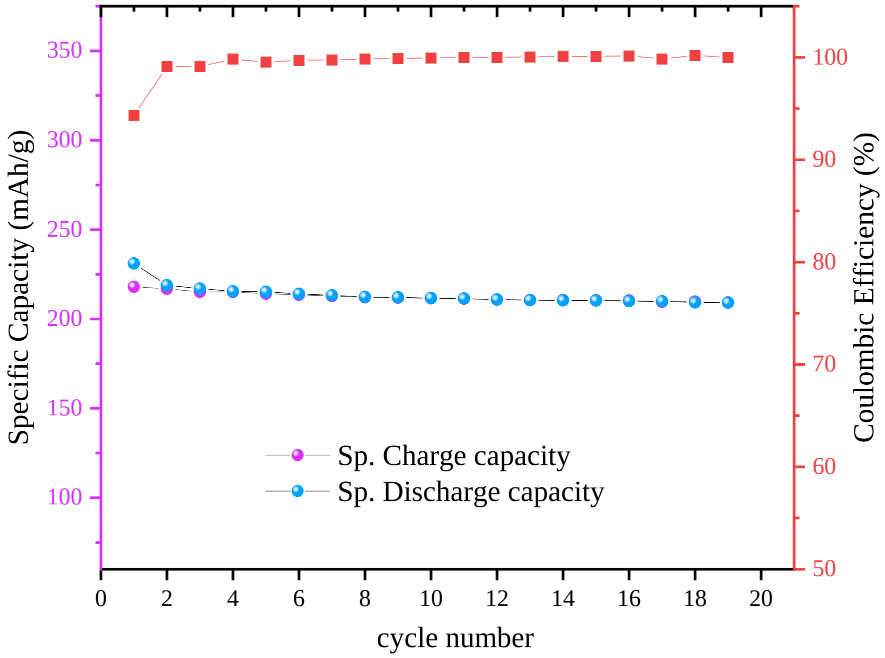

Figure S17. Cyclic capability of spent wood carbon monolith at a current density of 20 mA/g.

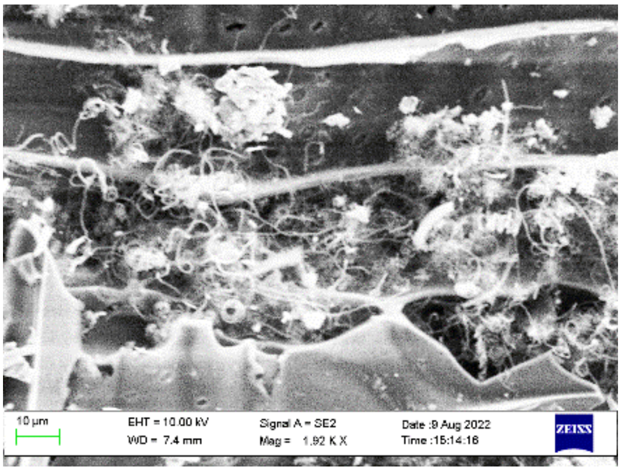

Figure S18. SEM image of fibrous carbon-filled channels in spent wood carbon monolith.

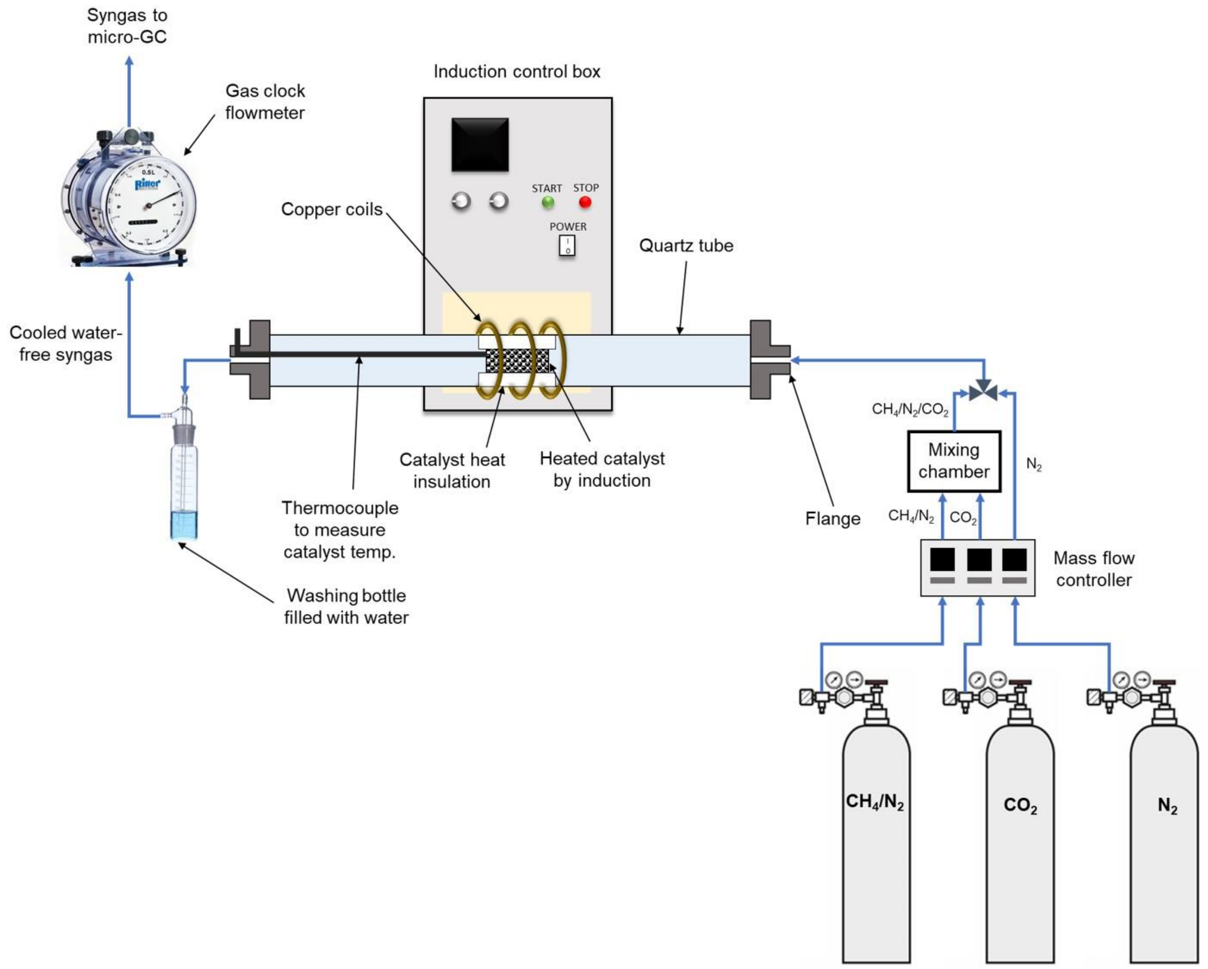

Figure S19. Schematic picture of the catalytic CH_4 dry reforming testing system.

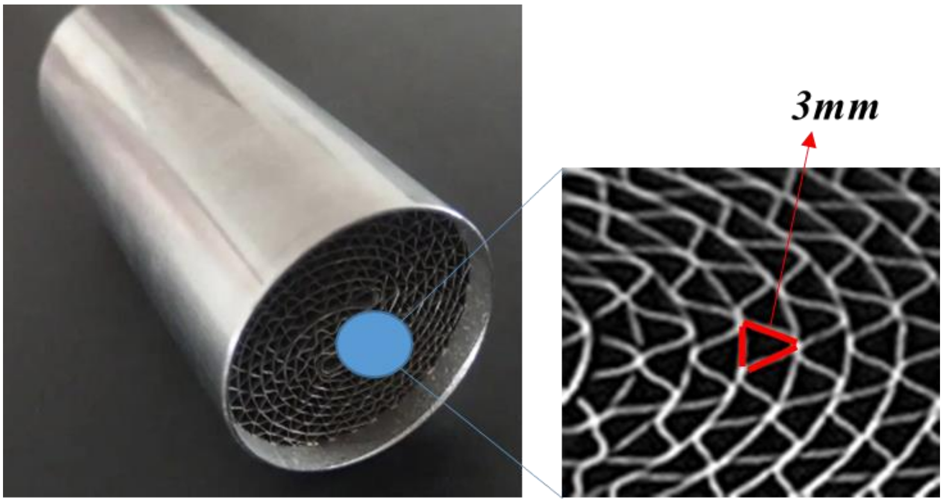

Figure S20. Picture of metallic monolith reactor with enlarged view.

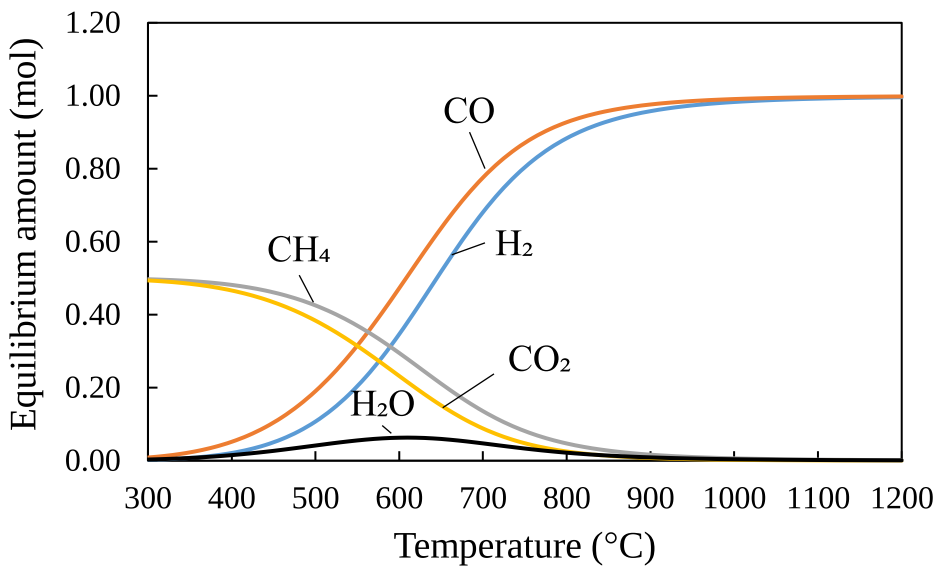

Figure S21. Equilibrium composition with temperatures of methane dry reforming in this study.

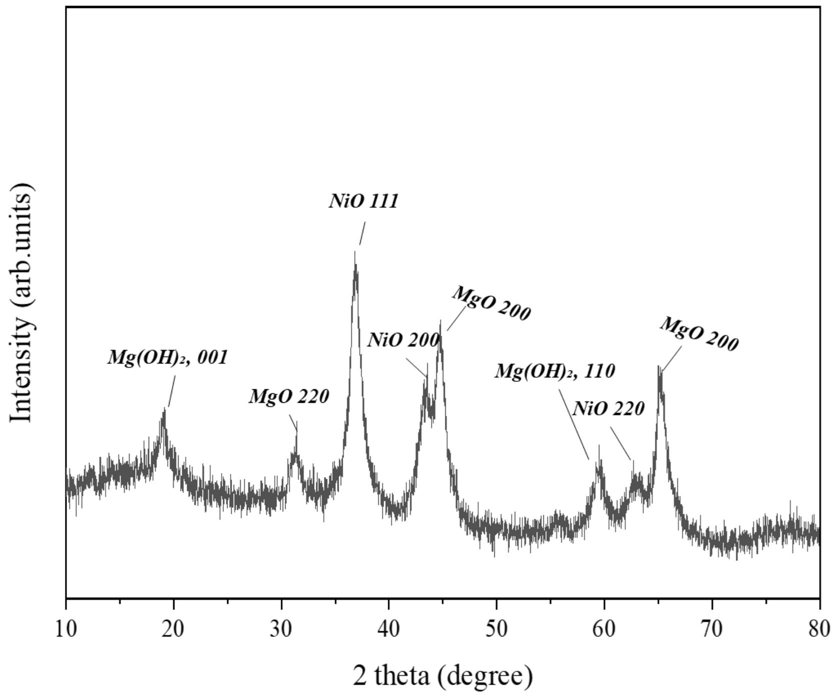

Figure S22. XRD pattern of the Ni/MgO washcoat.

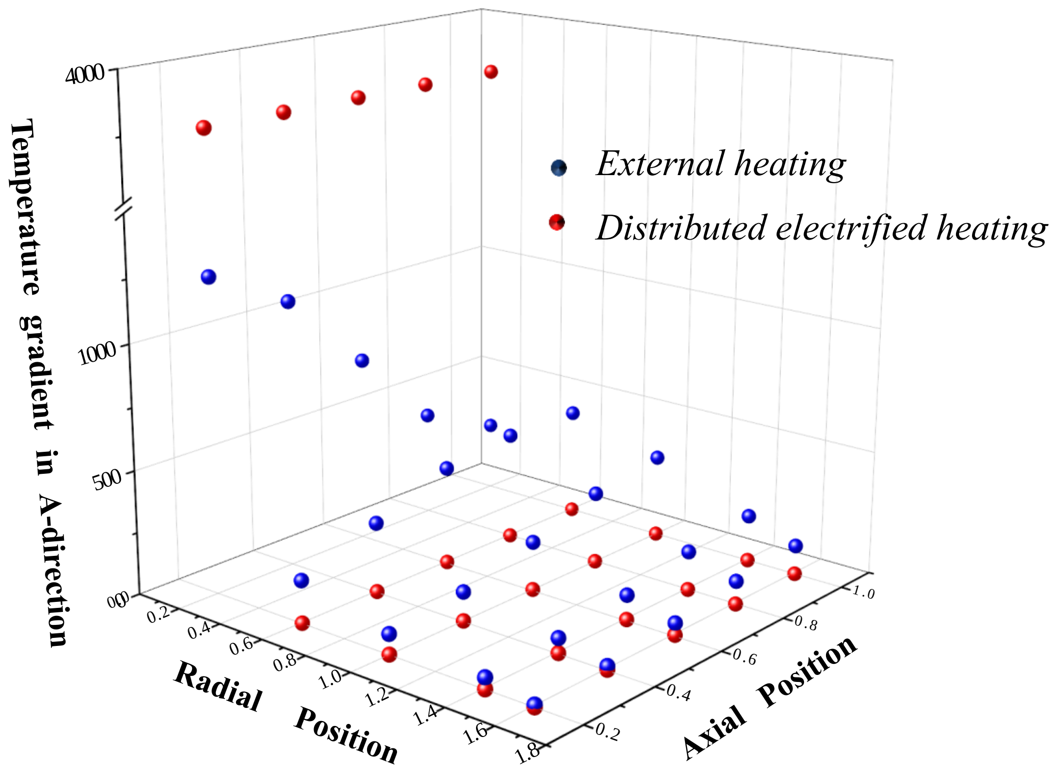

Figure S23. Temperature gradients in axial direction of different heating scenarios.

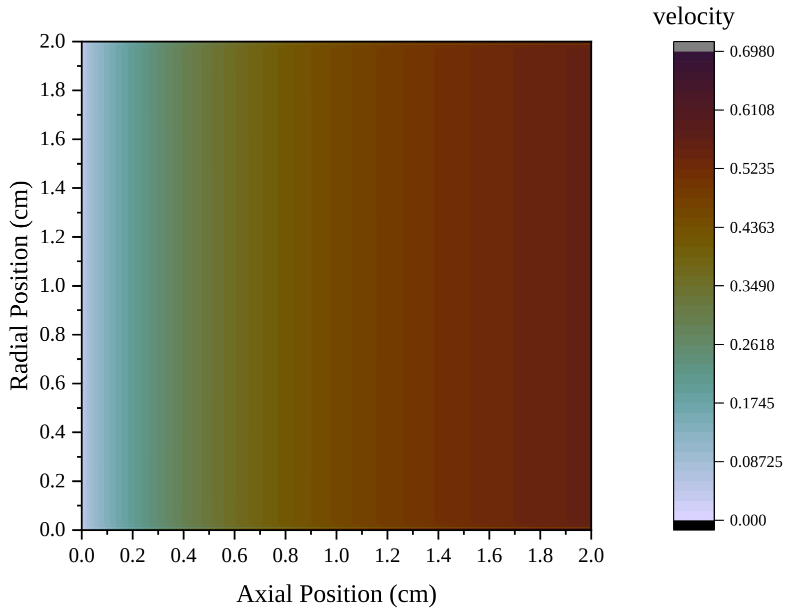

Figure S24. Space velocity distribution under distributed electrified heating within the reactor.

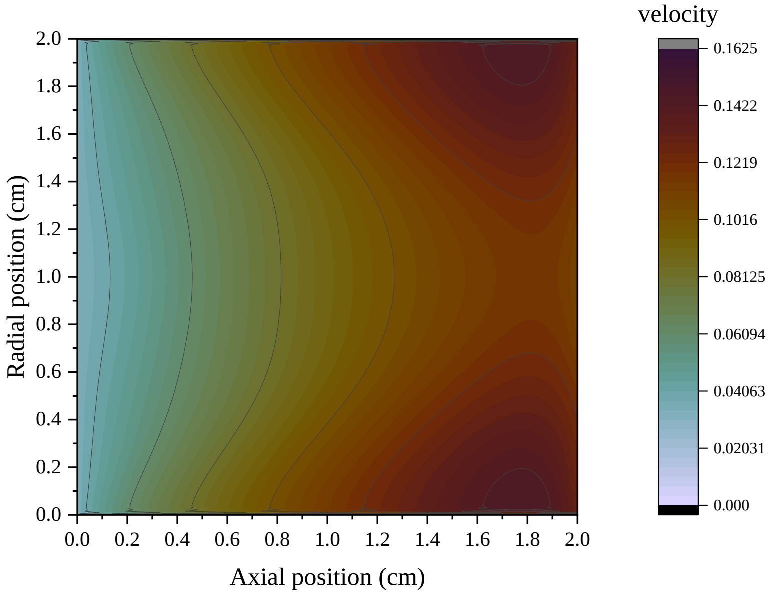

Figure S25. Space velocity distribution under external heating within the reactor.

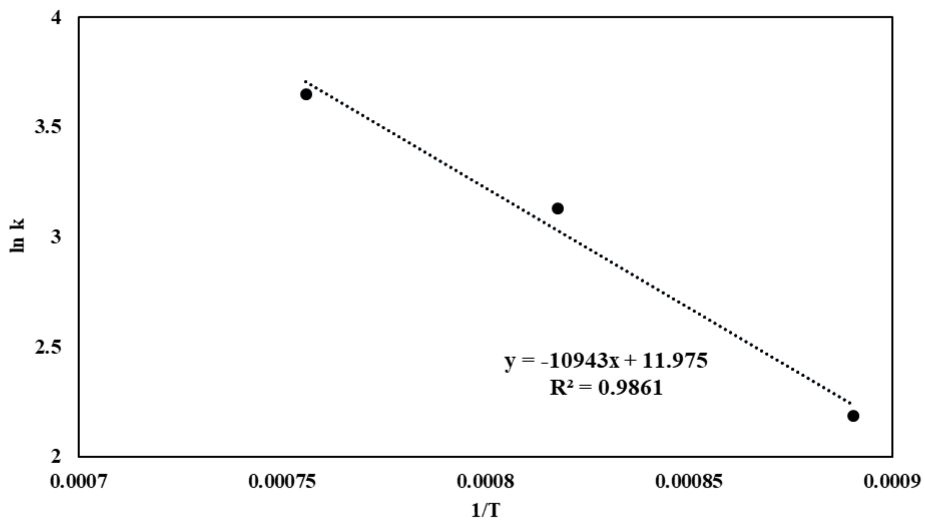

Figure S26. Obtained arrhenius plot for the CH_4 pyrolysis based on the experimental data.

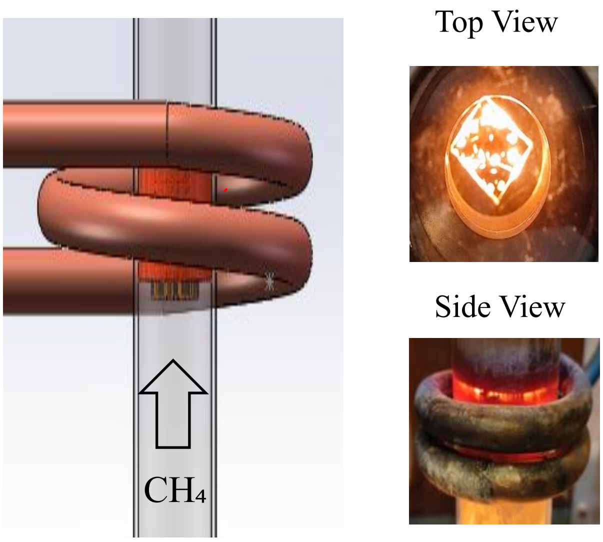

Figure S27. Top and side view photos of the reactor during the reaction.

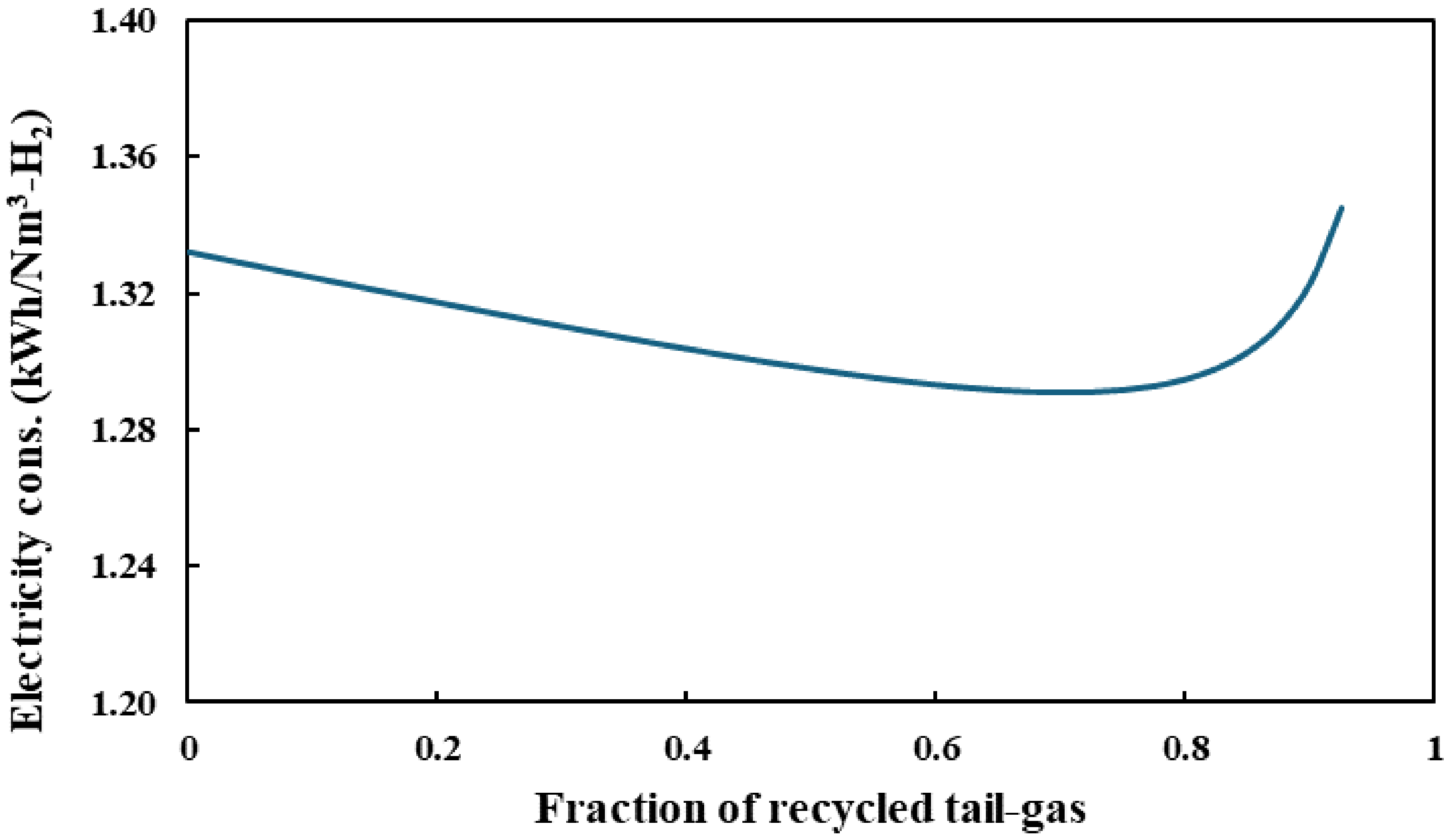

Figure S28. Effect of the amount of recycled tail gas to the specific energy demand of the production system.

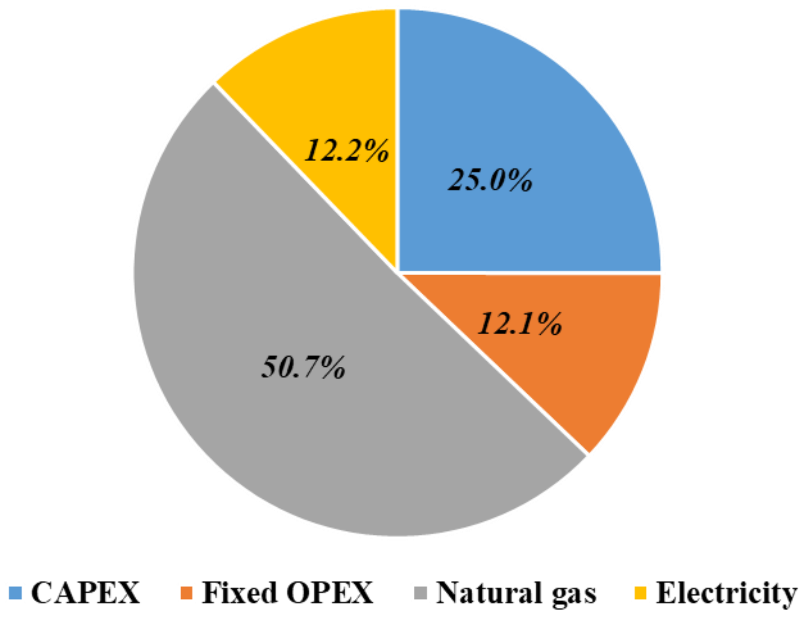

Figure S29. Breakdown of different costs of the distributed electrified heating-methane pyrolysis process (100kg/h natural gas, Natural gas price = 80 €/MWh, Electricity = 80 €/MWh). CAPEX: Capital expenditures; OPEX: Operating expenses.

Tables

Table S1. Specific surface areas and pore structure parameters for fresh and spent wood carbon monolith.

	**SSA m^2/g**	**V_{total} cm^3/g**	**V_{micro} cm^3/g**	**D_{mean} Å**
Fresh wood carbon monolith	326.7	0.16	0.002	7.65
Spent wood carbon monolith	7.5	0.017	0.002	7.64

Table S2. Flowrates used in this study and the corresponding space velocity.

Flowrate, mL/min	Space velocity, h^{-1}
100	750
200	1500
400	3000
800	6000

Table S3. Comparison of the different technologies for methane pyrolysis for hydrogen and/or carbon production.

Process shown by	***BASF***	***PARC***	***Monolith Materials***	***Hazer***	***UCSB***	***Present work***
Reactor	Moving bed	-	Plasma	Fluidized bed	Bubble Column	Wood carbon monolith
Catalyst	Carbon	Molten metal	No	Iron Ore	Molten metal/salts	No/carbon
GHSV (h^{-1})	>2000	>4000			<300	>3000
Temperature (°C)	1300	<950	2000	900	1065	1150
Hydrogen content at the reactor outlet	~92%	>90%	~95%	~92%	~95%	~100%
Carrier gas	Yes	Yes	Yes	Yes	Yes	No
Reactor pressure	Close to atmospheric pressure	-	Close to atmospheric pressure	Close to atmospheric pressure	Up to 5 bar	Close to atmospheric pressure
Solid Carbon	Carbon black	Graphite-like	Carbon black	Graphite-like	Graphite-like	Carbon fiber/Multi-wall carbon tube
Impurity content in carbon	Negligible	High, metal	Negligible	High, metal	High, metal	Negligible

Table S4. Ultimate and impurity analysis of the wood carbon monolith

**Element**	**Unit**	**Content**
C	% Ts	86.6
H	% Ts	4.4
N	% Ts	0.43
Impurity	Unit	Content
Al_2O_3	mg/kg Ts	340
CaO	mg/kg Ts	4200
K_2O	mg/kg Ts	1400
SiO_2	mg/kg Ts	1300

Table S5. CH_4 pyrolysis kinetic parameters in present work

E_a, kJ/mol	K_0, s^{-1}
91.01	1.59*10^5

Table S6. Boundary conditions for the numerical model.

External heating	Distributed electrified heating
x = 0 m: $\mathbf{u}_g = u_{in}$<br><br>x = 0.02 m: $p_g = 0$ Pa<br><br>y = 0 m & 0.02 m: $\mathbf{u}_g = 0$	x = 0 m: $\mathbf{u}_g = u_{in}$<br><br>u_{in} =0.004. 0.008, 0.017, 0.034 m/s<br><br>x = 0.02 m: $p_g = 0$ Pa<br><br>y = 0 m & 0.02 m: $\mathbf{u}_g = 0$
x = 0 m: $c_{CH_4} = 44.643\ \mathrm{mol/m^3}$,<br><br>$c_{H_2} = 0\ \mathrm{mol/m^3}$<br><br>$x$ = 0.02 m: $\mathbf{n} \cdot D_i \nabla c_i = 0$<br><br>$y$ = 0 m & 0.02 m: $-\mathbf{n} \cdot (-D_i \nabla c_i) = 0$	x = 0 m: $c_{CH_4} = 44.643\ \mathrm{mol/m^3}$,<br><br>$c_{H_2} = 0\ \mathrm{mol/m^3}$<br><br>$x$ = 0.02 m: $\mathbf{n} \cdot D_i \nabla c_i = 0$<br><br>$y$ = 0 m & 0.02 m: $-\mathbf{n} \cdot (-D_i \nabla c_i) = 0$
x = 0 m: $T_g = 25$ ℃<br><br>x = 0.02 m: $k_{g,eff} \frac{\partial T_g}{\partial x} = 0$<br><br>$y$ = 0 m & 0.02 m: $k_{g,eff} \frac{\partial T_g}{\partial y} = 0$	x = 0 m: $T_g = 25$ ℃<br><br>x = 0.02 m: $k_{g,eff} \frac{\partial T_g}{\partial x} = 0$<br><br>$y$ = 0 m & 0.02 m: $k_{g,eff} \frac{\partial T_g}{\partial y} = 0$
x = 0 m & 0.02 m: $k_{s,eff} \frac{\partial T_s}{\partial x} = \varepsilon\sigma(T_{amb}^4 - T_s^4)$<br><br>$y$ = 0 m & 0.02 m: $T_s = 850, 950\ ,1050, 1150$ ℃	$T_s = 850, 950, 1050, 1150$ ℃

Table S7. Main process parameters and assumptions in Aspen Plus and calculations.

Parameter	Modeling and assumption
Feedstock	Natural gas with a flowrate of 100 kg/h. The gas composition is based on the typical composition of natural gas in the European market [2] as follows (in mol%),<br>• CH_4 = 88.39<br>• CO_2 = 1.85<br>• N_2 = 0.95<br>• C_2H_6 = 7.07<br>• C_3H_8 = 1.37<br>• C_4H_{10} = 0.31<br>• C_5H_{12} = 0.03<br>• C_6H_{14} = 0.03
Pyrolysis reactor	Operating temperature = 1150 °C (based on the result of this study to achieve a full conversion of CH_4). The product composition and heat duty are calculates based on the Gibbs free energy minimization method in the RGIBBS reactor module. The total electricity consumption is calculated assuming a reactor efficiency of 88.3%, which corresponds to the estimated efficiency of an induction-heated reactor at the proposed scale (100 kg/h natural gas) according to the model developed by [21].
Cyclone	Simplified by using a separator module with a 100% carbon separation.
PSA	Black-box model considering only the energy consumption and target products' recovery and purity:<br>• Feed pressure = 30 bar [22]<br>• $P_{H2\ product}$ = 29 bar, $P_{PSA\ tail\ gas}$ = 1 bar [4]<br>• H_2 Recovery = 90%, H_2 Purity = 99.999% [4]
Multi-stage compressor	Two stages compression, cooling outlet temperature = 40 °C, discharge pressure = 30 bar.
Heat exchangers	Minimum temperature approach = 15 °C.

Table S8. Summary of financial assumptions for economic analysis

Parameter	Assumption
Currency	Euro 2022
Operating time or uptime	8000 h per year
Plant financing	100% equity financing
Internal rate of return	8%
Plant life	25 years
Construction period	1 year
Plant salvage value	No value
Government support	No financial support

Table S9. Input data for capital cost estimation

System	Base cost	Base scale	Scale unit	Scaling Factor	Ref.
Pyrolyzer unit					
Gas-fired furnace	28746 €2005	18.91	kg natural gas/h	0.7	[6]
Inductive heating element	1.27 M$2014	1	MW_{el}	1	[7]
Cyclone			Gas flow rate (1000 cfm)		[5]
HT Heat Exchanger (total installed)			A in m^2	Cost chart	[5]
Compressor	3300 €2006	5	kW_{el}	0.82	[23]
PSA unit	6.25 M€2002	0.294	kmol/s-purge gas	0.74	[8]
**Direct Plant Cost**	Cost estimation (% of TPEC)				[9]
Installation	39				
Instrumentation	13				
Piping	31				
Electrical installation	10				
Buildings	29				
Yard improvements	10				
Service facilities	55				

Land	6	
Direct Plant Cost (DPC)	293% of TPEC	DPC = Total Installed cost (TIC)
**Indirect capital cost (IDC)**		
Engineering and supervision	32	
Construction expenses	34	
Contractor's fee	18	
Contingency + Additional 20%	36 + 20% FCI	
Fixed capital Investment (FCI)	DPC + IDC = 413% of TPEC + 0.83	
Working capital (WC)	74	
**Total Capital Investment (TCI)**	FCI + WC = 487% of TPEC + 0.83	

Table S10. Input data for operating cost estimation

Specification	Cost estimation		
Fixed operating cost			
Labor	2.5% TCI		
Maintanance	1% TCI		
Insurance	0.5% TCI		
Plant overhead	10% operating labor		
Misc. OPEX	10% operating labor		
Variable operating cost			
Items	Value	Unit	Ref.
Natural gas	20-120	€/MWh - LHV	EU weighted average price 2022 [25]
Electricity	20-120	€/MWh	[26]
Cooling water	0.8	€/m^3	[27]
Carbon nanofibers	20 - >200	€/kg	[28]
District heating	40	€/MWh	[29]

Table S11. Summary of TEA on hydrogen production from methane from the literature

Technology	Plant capacity	Case scenario	Carbon sales	LCOH	Ref.
Plasma-heated CH_4 pyrolysis	6.7 kt/a H_2	Various energy requirement of the plasma reactor (1.31-2.8 kWh/Nm^3 H_2)；<br>Natural gas price of 25 €/MWh<br>Electricity price of 64 €/MWh	0.23-1.62 €/kg	0-4.8 €/kg H_2	[30]
Molten gallium bubble column reactor	21 kt/a H_2	Heat supplied by electricity;<br>Natural gas price 30 €/MWh<br>Electricity price 76.36 €/MWh	0.3 €/kg	3.16 €/kg H_2	[31]
electron beam plasma methane pyrolysis	72 kt/a H_2	Electric supplied by renewable energy input;<br>Natural gas price 10 €/MWh<br>Electricity 60 €/MWh	0.1 €/kg	2.55 €/kg H_2	[32]
Molten salt bubble column reactor natural gas pyrolysis	21.6 kt/a H_2	H_2 sales price in the case of no revenue from the produced carbon;<br>Natural gas price 23.4 €/MWh<br>Electicity 60 €/MWh	NA	2.38-2.62 €/kg H_2	[33]
EAF-heated molten metals methane pyrolysis	200 kt/a H_2	Reaction temperature of 1500 °C;<br>Natural gas 9.5 €/MWh<br>Electricity 42 €/MWh	NA	1.6 €kg H_2	[34]
hydrogen production by sorption enhanced steam	500 kt/a CH_4	Reaction temperature of 600 °C;	NA	2.08 €/kg H_2	[35]

methane reforming (SE-SMR) processes		Natural gas price 18 €/MWh,<br>Electricity 187 €/MWh			
SE-SMR with CCS	500 kt/a CH_4		NA	2.22 €/kg H_2	
Distributed electrified heating-natural gas pyrolysis	0.8 kt/a CH_4	Natural gas price = 80 €/MWh<br>Electricity = 80 €/MWh	2 €/kg	2.71 €/kg H_2	This study
	0.8 kt/a CH_4	Natural gas price = 80 €/MWh<br>Electricity = 80 €/MWh	5 €/kg	Negative	

Note: CO_2 tax is not considered in the above cases

Reference:

[1] S.R. Patlolla, K. Katsu, A. Sharafian, K. Wei, O.E. Herrera, W. Mérida, A review of methane pyrolysis technologies for hydrogen production, Renewable and Sustainable Energy Reviews, 181 (2023) 113323.
[2] Swedegas, Gaskvaliteten följs noggrant.
[3] C. Jensen, M.S. Duyar, Thermodynamic Analysis of Dry Reforming of Methane for Valorization of Landfill Gas and Natural Gas, Energy Technology, 9 (2021) 2100106.
[4] C.A. Grande, PSA technology for H2 separation, Hydrogen Science and Engineering: Materials, Processes, Systems and Technology, (2016) 489-508.
[5] D.E. Garrett, Chemical engineering economics, Springer Science & Business Media2012.
[6] N. Inc., Equipment Design and Cost Estimation for Small Modular Biomass Systems, Synthesis Gas Cleanup, and Oxygen Separation Equipment; Task 1: Cost Estimates of Small Modular Systems, Office of Scientific and Technical Information (OSTI), 2006.
[7] A. Bera, T. Babadagli, Status of electromagnetic heating for enhanced heavy oil/bitumen recovery and future prospects: A review, Appl Energ, 151 (2015) 206-226.
[8] T. Kreutz, R. Williams, S. Consonni, P. Chiesa, Co-production of hydrogen, electricity and CO2 from coal with commercially ready technology. Part B: Economic analysis, International Journal of Hydrogen Energy, 30 (2005) 769-784.
[9] M.S. Peters, K.D. Timmerhaus, R.E. West, K. Timmerhaus, R. West, Plant design and economics for chemical engineers, McGraw-hill New York1968.
[10] Y. Wang, G. Sun, J. Dai, G. Chen, J. Morgenstern, Y. Wang, S. Kang, M. Zhu, S. Das, L. Cui, A high‐performance, low‐tortuosity wood‐carbon monolith reactor, Advanced Materials, 29 (2017) 1604257.
[11] Y. Sun, Z. He, Y. Wei, G. Liu, R. Liu, J. Hu, H. Liu, X. Zhang, G. Yuan, Wood‐Derived Monolithic Carbon Materials and Their Functional Applications, CLEAN–Soil, Air, Water, 50 (2022) 2100420.
[12] Y. Jin, H. Yang, S. Guo, Z. Shi, T. Han, R. Gond, P.G. Jönsson, W. Yang, Carbon and H2 recoveries from plastic waste by using a metal-free porous biocarbon catalyst, J Clean Prod, 404 (2023) 136926.
[13] A. Abánades, E. Ruiz, E. Ferruelo, F. Hernández, A. Cabanillas, J. Martínez-Val, J. Rubio, C. López, R. Gavela, G. Barrera, Experimental analysis of direct thermal methane cracking, International journal of hydrogen energy, 36 (2011) 12877-12886.
[14] Z. Jia, K. Kou, M. Qin, H. Wu, F. Puleo, L.F. Liotta, Controllable and large-scale synthesis of carbon nanostructures: A review on bamboo-like nanotubes, Catalysts, 7 (2017) 256.
[15] B. Fidalgo Fernández, Y. Fernández Díez, A. Domínguez Padilla, J.J. Pis Martínez, J.Á. Menéndez Díaz, Microwave-assisted pyrolysis of CH4/N2 mixtures over activated carbon, (2008).
[16] X. Zeng, D. Fu, H. Sheng, S. Xie, X. Li, Q. Hu, J. Zou, Growth and morphology of carbon nanostructures by microwave-assisted pyrolysis of methane, Physica E: Low-dimensional Systems and Nanostructures, 42 (2010) 2103-2108.
[17] M. Dadsetan, M.F. Khan, M. Salakhi, E.R. Bobicki, M.J. Thomson, CO2-free hydrogen production via microwave-driven methane pyrolysis, International Journal of Hydrogen Energy, 48 (2023) 14565-14576.
[18] K.K. Lee, G.Y. Han, K.J. Yoon, B.K. Lee, Thermocatalytic hydrogen production from the methane in a fluidized bed with activated carbon catalyst, Catal Today, 93 (2004) 81-86.
[19] A. Domínguez Padilla, B. Fidalgo Fernández, Y. Fernández Díez, J.J. Pis Martínez, J.Á. Menéndez Díaz, Microwave-assisted catalytic decomposition of methane over activated carbon for CO2-free hydrogen production, (2007).

[20] X.-X. He, J.-H. Zhao, W.-H. Lai, R. Li, Z. Yang, C.-m. Xu, Y. Dai, Y. Gao, X.-H. Liu, L. Li, Soft-carbon-coated, free-standing, low-defect, hard-carbon anode to achieve a 94% initial Coulombic efficiency for sodium-ion batteries, ACS Applied Materials & Interfaces, 13 (2021) 44358-44368.
[21] M.R. Almind, S.B. Vendelbo, M.F. Hansen, M.G. Vinum, C. Frandsen, P.M. Mortensen, J.S. Engbæk, Improving performance of induction-heated steam methane reforming, Catal Today, 342 (2020) 13-20.
[22] F. Pruvost, S. Cloete, J.H. Cloete, C. Dhoke, A. Zaabout, Techno-Economic assessment of natural gas pyrolysis in molten salts, Energ Convers Manage, 253 (2022) 115187.
[23] G.D. Marcoberardino, D. Vitali, F. Spinelli, M. Binotti, G. Manzolini, Green hydrogen production from raw biogas: A techno-economic investigation of conventional processes using pressure swing adsorption unit, Processes, 6 (2018) 19.
[24] I.E. Agency, World energy outlook 2020, OECD Publishing2020.
[25] E. (2023a). Natural gas price statistics. .
[26] E. (2023b). Electricity price statistics.
[27] R. Turton, R.C. Bailie, W.B. Whiting, J.A. Shaeiwitz, Analysis, synthesis and design of chemical processes, Pearson Education2008.
[28] J. Riley, C. Atallah, R. Siriwardane, R. Stevens, Technoeconomic analysis for hydrogen and carbon Co-Production via catalytic pyrolysis of methane, International Journal of Hydrogen Energy, 46 (2021) 20338-20358.
[29] F. Levihn, L. Linde, K. Gustafsson, E. Dahlen, Introducing BECCS through HPC to the research agenda: The case of combined heat and power in Stockholm, Energy reports, 5 (2019) 1381-1389.
[30] A.R. da Costa Labanca, Carbon black and hydrogen production process analysis, International Journal of Hydrogen Energy, 45 (2020) 25698-25707.
[31] B.J. Leal Pérez, J.A. Medrano Jiménez, R. Bhardwaj, E. Goetheer, M. van Sint Annaland, F. Gallucci, Methane pyrolysis in a molten gallium bubble column reactor for sustainable hydrogen production: Proof of concept & techno-economic assessment, International Journal of Hydrogen Energy, 46 (2021) 4917-4935.
[32] F. Kerscher, A. Stary, S. Gleis, A. Ulrich, H. Klein, H. Spliethoff, Low-carbon hydrogen production via electron beam plasma methane pyrolysis: Techno-economic analysis and carbon footprint assessment, International Journal of Hydrogen Energy, 46 (2021) 19897-19912.
[33] F. Pruvost, S. Cloete, J. Hendrik Cloete, C. Dhoke, A. Zaabout, Techno-Economic assessment of natural gas pyrolysis in molten salts, Energ Convers Manage, 253 (2022) 115187.
[34] B. Parkinson, J.W. Matthews, T.B. McConnaughy, D.C. Upham, E.W. McFarland, Techno‐Economic Analysis of Methane Pyrolysis in Molten Metals: Decarbonizing Natural Gas, Chemical Engineering & Technology, 40 (2017) 1022-1030.
[35] Y. Yan, V. Manovic, E.J. Anthony, P.T. Clough, Techno-economic analysis of low-carbon hydrogen production by sorption enhanced steam methane reforming (SE-SMR) processes, Energ Convers Manage, 226 (2020) 113530.
